# The *Helicobacter pylori* J99 *jhp0106* Gene, under the Control of the CsrA/RpoN Regulatory System, Modulates Flagella Formation and Motility

**DOI:** 10.3389/fmicb.2017.00483

**Published:** 2017-03-28

**Authors:** Cheng-Yen Kao, Jenn-Wei Chen, Shuying Wang, Bor-Shyang Sheu, Jiunn-Jong Wu

**Affiliations:** ^1^Department of Biotechnology and Laboratory Science in Medicine, School of Biomedical Science and Engineering, National Yang Ming UniversityTaipei, Taiwan; ^2^Center of Infectious Disease and Signaling Research, National Cheng Kung UniversityTainan, Taiwan; ^3^Department of Microbiology and Immunology, College of Medicine, National Cheng Kung UniversityTainan, Taiwan; ^4^Department of Internal Medicine, College of Medicine, National Cheng Kung University Hospital, National Cheng Kung UniversityTainan, Taiwan; ^5^Department of Internal Medicine, Tainan Hospital, Ministry of Health & WelfareTainan, Taiwan

**Keywords:** flagella, glycosylation, motility, CsrA, *jhp0106*

## Abstract

CsrA has been shown to positively control the expression of flagella-related genes, including *flaA* and *flaB*, through regulating expression of an alternative sigma factor RpoN in *Helicobacter pylori* J99. Here, we aimed to characterize the CsrA regulatory system by comparative transcriptomic analysis carried out with RNA-seq on strain J99 and a *csrA* mutant. Fifty-three genes in the *csrA* mutant were found to be differentially expressed compared with the wild-type. Among CsrA-regulated genes, *jhp0106*, with unclear function, was found located downstream of *flaB* in the J99 genome. We hypothesized that *flaB*-*jhp0106* is in an operon under the control of RpoN binding to the *flaB* promoter. The RT-qPCR results showed the expression of *jhp0106* was decreased 76 and 92% in the *csrA* and *rpoN* mutants, respectively, compared to the wild-type. Moreover, mutations of the RpoN binding site in the *flaB* promoter region resulted in decreased expression of *flaB* and *jhp0106* and deficient motility. Three-dimensional structure modeling results suggested that Jhp0106 was a glycosyltransferase. The role of *jhp0106* in *H. pylori* was further investigated by constructing the *jhp0106* mutant and revertant strains. A soft-agar motility assay and transmission electron microscope were used to determine the motility and flagellar structure of examined strains, and the results showed the loss of motility and flagellar structure in *jhp0106* mutant J99. In conclusion, we found *jhp0106*, under the control of the CsrA/RpoN regulatory system, plays a critical role in *H. pylori* flagella formation.

## Introduction

*Helicobacter pylori* is a highly prevalent human pathogen that colonizes roughly 50% of the world's population. Persistent infection with *H. pylori* increases the risk of developing gastroduodenal diseases, including chronic gastritis, gastric and duodenal ulcer, and gastric adenocarcinoma (Parsonnet et al., 1991; Graham et al., 1993; Ahmad et al., 2003). Motility of *H. pylori* mediated by flagella has been shown to be critical for the cells to establish initial colonization and achieve dense colonization and severe pathological outcomes in patients (Eaton et al., 1996; Ottemann and Lowenthal, 2002; Kao et al., 2012a, 2016). Despite intensive research in the role of motility in *H. pylori* pathogenesis, the complex regulatory network that modulates the expression of flagellar genes in *H. pylori* is still not fully understood.

*H. pylori* has five to seven polar, sheathed flagella, which are composed of three main structures: the basal body, hook and filament (Lertsethtakarn et al., 2011). Flagellar related genes are divided into three classes, governed by the housekeeping sigma factor σ^80^ (RpoD, regulating class I genes), the alternative sigma factors σ^54^ (RpoN, regulating class II genes), and σ^28^ (FliA, regulating class III genes) (Niehus et al., 2004; Kao et al., 2016). The flagellar filament consists of two flagellin proteins, FlaA (the major constituent) and FlaB (Kostrzynska et al., 1991; Suerbaum et al., 1993). *H. pylori* flagellin proteins are synthesized, then post-translationally modified intracellularly by glycosylation with a nine carbon pseudaminic acid sugar derivative that resembles sialic acid (Schirm et al., 2003; Logan, 2006). The enzymes of the pseudaminic acid biosynthetic pathway in *H. pylori*, in order, are PseB, PseC, PseH, PseG, and PseI (Schirm et al., 2003; Menard et al., 2014), and the glycosylation process is essential for assembly of functional flagellar filaments and consequent bacterial motility (Schoenhofen et al., 2006).

CsrA was identified as a post-transcriptional regulator of glycogen biosynthesis, motility, biofilm formation and bacterial virulence in *E. coli*, acting as an RNA binding protein on its target mRNA and thus affecting its stabilization or translation (Romeo et al., 1993, 2013; Liu et al., 1995; Liu and Romeo, 1997; Wang et al., 2005; Jonas et al., 2008). In *H. pylori* strain J99, CsrA regulates flagella formation by controlling RpoN expression, and it thereby affects bacterial motility (Kao et al., 2014). Although the decrease of FlaA/FlaB partially explains the non-flagellated phenotype of the *csrA* mutant observed by transmission electron microscopy (TEM) (Kao et al., 2014), other regulators or mechanisms may be involved in the CsrA regulatory system. In this study, we aimed to characterize the CsrA regulatory system by comparative transcriptomic analysis carried out with RNA-seq on *H. pylori* strain J99 and a *csrA* mutant. We demonstrated that Jhp1006, a putative glycosyltransferase involved in *H. pylori* J99 flagella formation and motility, is under the control of CsrA/RpoN.

## Materials and methods

### Bacterial strains and growth conditions

The bacterial strains and plasmids used in this study are described in Table 1. *H. pylori* cells were grown on CDC anaerobic blood agar (BBL, Microbiology Systems, Cockeysville, MD) or in Brucella broth containing 10% (v/v) horse serum (Gibco BRL, Life Technologies, Rockville, MD) at 37°C in microaerophilic conditions (5% O_2_, 10% CO_2_ and 85% N_2_). *E. coli* was grown on Luria-Bertani (LB) (BD Biosciences, San Jose, CA) agar or in broth. Bacteria harboring antibiotic resistance determinants were grown in the presence of the appropriate antibiotics at the following concentrations: ampicillin (Amp, 100 μg ml^−1^); chloramphenicol (Cm, 25 μg ml^−1^ for *E. coli*, 10 μg ml^−1^ for *H. pylori*); kanamycin (Km, 50 μg ml^−1^ for *E. coli*, 10 μg ml^−1^ for *H. pylori*). All strains were stored at −80°C in Brain-Heart Infusion (BHI) broth (*H. pylori*) or LB broth (*E. coli*) containing 20% (v/v) glycerol until testing.

**Table 1:** **Strains and plasmids used in this study**.

**Strain or plasmid**	**Relevant genotype or description**	**Reference or source**
***E. coli*** **STRAIN**		
DH5α	F^−^Ψ *80dlacZΔM15 Δ(lacZYA-argF) U169 hsdR17 recA1 thi-1 relA1*	Laboratory stock
***H. pylori*** **STRAIN**		
J99	Isolated from patient with duodenal ulcer; motile	Alm et al., 1999
SW835	*csrA* mutant J99; non-motile; Cm^r^	Kao et al., 2014
SW836	*csrA* revertant, derived from SW835; motile	Kao et al., 2014
SW837	*rpoN* mutant J99; non-motile; Cm^r^	Kao et al., 2014
SW838	*rpoN* revertant, derived from SW837; motile	This study
SW853	Mutation of RpoN binding site of the *flaB* promoter (CG to AA, GG to AA)	This study
SW854	Mutation of RpoN binding site of the *flaB* promoter (GG to AA)	This study
SW855	Mutation of RpoN binding site of the *flaB* promoter (deletion)	This study
SW856	Mutation of RpoN binding site of the *flaB* promoter (*cat* cassette insertion); Cm^r^	This study
SW857	*flaB* promoter revertant, derived from SW856	This study
SW858	*flaB* revertant, derived from SW859	This study
SW859	*flaB* mutant J99 (*flaB*/*cat* in opposite direction); Cm^r^	This study
SW860	*flaB* revertant, derived from SW861	This study
SW861	*flaB* mutant J99 (*flaB*/*cat* in same direction); Cm^r^	This study
SW862	*jhp0106* revertant, derived from SW863	This study
SW863	*jhp0106* mutant J99; non-motile; Km^r^	This study
SW866	*flaA* mutant J99; non-motile; Km^r^	This study
SW868	*flaA*/*flaB* double mutant J99, derived from SW861; non-motile; Cm^r^, Km^r^	This study
**PLASMID**		
pUC18	A general cloning vector with *lacZ* selection; Amp^r^	Invitrogen
pMW758	pUC18 containing the *rpoN* fragment; Amp^r^	Kao et al., 2014
pMW801	pUC18 containing the *jhp0106* fragment; Amp^r^	This study
pMW802	pMW801 containing a *aph*(*3'*)-*III* cassette inserted into the *jhp0106* fragment; Amp^r^, Km^r^	This study
pMW810	pMW814 with mutations of RpoN binding site of the *flaB* promoter (CG to AA, GG to AA); Amp^r^	This study
pMW811	pMW814 with mutations of RpoN binding site of the *flaB* promoter (GG to AA); Amp^r^	This study
pMW812	pMW813 containing a *cat* cassette inserted into the *flaB* promoter fragment; Amp^r^, Cm^r^	This study
pMW813	pMW814 containing a *Nae*I cutting site and 46 bp removal in the *flaB* promoter fragment; Amp^r^	This study
pMW814	pUC18 containing the *flaB* promoter fragment; Amp^r^	This study
pMW815	pMW817 containing a *cat* cassette inserted into the *flaB* fragment (*flaB*/*cat* in opposite direction); Amp^r^, Cm^r^	This study
pMW816	pMW817 containing a *cat* cassette inserted into the *flaB* fragment (*flaB*/*cat* in same direction); Amp^r^, Cm^r^	This study
pMW817	pUC18 containing the *flaB* fragment; Amp^r^	This study
pMW833	pUC18 containing the *flaA* fragment; Amp^r^	This study
pMW834	pMW833 containing a *aph*(*3'*)-*III* cassette inserted into the *flaA* fragment; Amp^r^, Km^r^	This study
Vector78	A vector containing *cat* cassette inserted in *Hinc*II site; Cm^r^	Wang and Taylor, 1990
pBHP489k	A vector containing *aph*(*3'*)-*III* cassette inserted in *Cla*I site; Km^r^	Lee et al., 1997

*cat, chloramphenicol acetyltransferase; Amp^r^, ampicillin resistant; Cm^r^, chloramphenicol resistant; Km^r^, kanamycin resistant*.

### Cell line and cell culture

The human gastric carcinoma cell line AGS (purchased from ATCC; American Type Culture Collection, Manassas, VA, USA) was grown in Ham's F-12 medium (Invitrogen Life Technologies, Rockville, MD) supplemented with 10% (v/v) fetal bovine serum (Gibco BRL) in an atmosphere consisting of 5% CO_2_ at 37°C. The human gastric epithelial immortalized GES-1 cells (a gift from Prof. Wei-Lun Chang, National Cheng Kung University Hospital) were grown in RPMI 1640 medium (Invitrogen Life Technologies) supplemented with 10% (v/v) fetal bovine serum in an atmosphere consisting of 5% CO_2_ at 37°C.

### DNA techniques

Mini Qiagen columns and a QiaAmp DNA extraction kit (Qiagen, Valencia, CA, USA) were used for plasmid and chromosomal DNA extraction. PCR was carried out according to the manufacturer's instruction using Taq polymerase (Promega, Madison, WI, USA).

### RNA-seq library preparation and sequencing

*H. pylori* cells were grown on CDC plates in microaerophilic conditions for 36 h, then transferred to 100 ml Brucella broth containing 10% (v/v) horse serum at an optical density (OD) of 0.2 and incubated with shaking (150 rpm, to reduce cell aggregation) for 18 h in microaerophilic conditions. Mini Qiagen columns and a Qiagen RNAeasy mini kit (Qiagen, Valencia, CA) were used for RNA extraction. Ribosomal RNA was depleted according to the manufacturer's instruction using Bacteria Minus™ Transcriptome Isolation Kit (Invitrogen Life Technologies). The Applied Biosystems SOLiD™ Total RNA-Seq kit was used to generate the cDNA template library. The SOLiD™ EZ Bead system (Invitrogen Life Technologies) was used to perform emulsion clonal bead amplification to generate bead templates for SOLiD™ platform sequencing. Samples were sequenced on the 5500XL SOLiD™ platform. The 50-base short read sequences produced by the 5500XL SOLiD™ sequencer were first run through SOLiD Accuracy Enhancement Tool (SAET) to improve color call accuracy, then were mapped in color space using SOLiD™ LifeScope™ software version 2.5 using default parameters against the *H. pylori* J99 reference genome (NCBI accession number, NC_000921). The BAM file from LifeScope™ was performed the analysis of gene expression with Partek software package. The gene expression from each sample was then tested for statistical differences using one-way ANOVA at 5% confidence level. The complete set of RNA-seq files has been deposited in Gene expression omnibus (GEO), NCBI (https://www.ncbi.nlm.nih.gov/geo/, accession number GSE95006). The fold change of each gene was measured as the mean of three independent experiments. Gene expression of the *csrA* mutant compare to wild-type J99 with a fold change > 1.5-fold was selected and confirmed by RT-qPCR.

### Preparation of cDNA from *H. pylori*

*H. pylori* cells were grown on CDC plates in microaerophilic conditions for 36 h, then transferred to 100 ml Brucella broth containing 10% (v/v) horse serum at an OD of 0.2 and incubated with shaking (150 rpm) for 18 h in microaerophilic conditions. RNA extraction and reverse transcription PCR were described previously (Kao et al., 2012b). Thirty microliters of culture media were centrifuged at 1,000 × g for 5 min at 4°C and then washed with ice-cold 0.2 M sodium acetate buffer (pH 5.5) twice. The bacterial pellet was then re-suspended in 600 μl acetate buffer (20 mM sodium acetate, 1 mM EDTA, and 0.5% (w/v) SDS), and 600 μl acid-phenol (pH 4.5) was added to isolate bacterial RNA. The sample was incubated at 65°C for 10 min, and centrifuged at 12,000 × g for 10 min to collect the supernatant. After isopropanol precipitation, the sample was treated with DNase I (Promega) at 37°C for 2 h. Finally, phenol/chloroform was used to extract total RNA, and the sample was dissolved in diethylpyrocarbonate (DEPC)-treated deionized water and stored at −80°C until used. The RNA was quantified at an absorbance of 260 nm. Random hexamers (Mission biotech, Taiwan) and MMLV reverse transcriptase (Promega) were used to generate cDNA from 1 μg of total RNA, and the cDNA was stored at −20°C until testing.

### Real-time quantitative RT-PCR (RT-qPCR)

The primers used for RT-qPCR are listed in Table S1. RNA quantification was carried out by RT-qPCR with a KAPA PROBE FAST Universal 2 x qPCR Master Mix (KAPA Biosystems Inc., Woburn, MA) specifically adapted for one-step RT-qPCR in glass capillaries using a Light Cycler instrument (Roche Diagnostics, Indianapolis, IN). Cycling conditions were as follows: activation of the polymerase for 10 min at 95°C, followed by 40 cycles of denaturation at 95°C for 20 s, annealing at 60°C for 1 min, and elongation at 72°C for 15 s. Fluorescence was detected at the end of each extension step, and the Cp values were calculated by the LightCycler 1.5 software.

### Construction of the mutants and revertants

The primers used in this study are listed in Table S1. In order to construct a revertant strain from *rpoN* mutant J99 (SW837), the plasmid containing the *rpoN* fragment, pMW758 (Kao et al., 2014), was transformed into SW837 to generate a revertant strain containing the wild-type *rpoN*. In brief, after natural transformation (Haas et al., 1993), *H. pylori* was grown on CDC anaerobic blood agar without antibiotic for 3 days. Colonies were picked and subcultured on CDC anaerobic blood agar without antibiotic and Brucella agar plate containing 10% (v/v) horse serum and Cm (10 μg ml^−1^) at the same time and incubated at 37°C in microaerophilic conditions for 3 days. Colonies that lost the ability to grow on a Cm-containing plate (that only grew on CDC anaerobic blood agar) were considered as the revertant strain and were verified by PCR-sequencing and motility assay.

For *flaB* mutant construction, the 1,175 bp *flaB* fragment obtained from *H. pylori* J99 genomic DNA and PCR with flaB-Mut-1 and flaB-Mut-2 primers, was digested by *Pst*I and *Kpn*I and ligated to the plasmid pUC18 to generate plasmid pMW817. A chloramphenicol acetyltransferase cassette (*cat* cassette) containing 806 bp was obtained from plasmid vector 78, cut with *Hinc*II and inserted into plasmid pMW817 digested with *Hinc*II. Two plasmids were designated as plasmid pMW815 (*flaB*/*cat* in opposite transcriptional orientation) and pMW816 (*flaB*/*cat* in same transcriptional orientation), respectively, and transformed into J99. Cm (10 μg ml^−1^) was used to select for the *flaB* mutants, SW859 and SW861, generated from chromosomal *flaB* double cross-over with plasmids pMW815 and pMW816, respectively. Plasmid pMW817 was then transformed into SW859 and SW861 to generate a revertant strains SW858 and SW860, respectively.

For *jhp0106* mutant construction, the 1,004 bp *jhp0106* fragment, obtained from J99 genomic DNA and PCR with jhp0106-Mut-1 and jhp0106-Mut-2 primers, was digested with *EcoR*I and ligated into the plasmid pUC18 to generate plasmid pMW801. A kanamycin resistance cassette (*aph*(*3*′)-*III* cassette) was obtained from plasmid vector pBHP489K, cut with *Cla*I and inserted into the plasmid pMW801 digested with *Bsm*I. This plasmid was designated as plasmid pMW802 and transformed into J99. Km (10 μg ml^−1^) was used to select for the *jhp0106* mutant, SW863. In order to construct a *jhp0106* revertant from SW863, the plasmid pMW801, was transformed into SW863 to generate SW862 containing wild-type *jhp0106*.

For *flaA* and *flaA*/*flaB* mutants construction, the 1,283 bp *flaA* fragment obtained from *H. pylori* J99 genomic DNA and PCR with FlaA-Mut-1 and FlaA-Mut-2 primers, was digested by *Pst*I and *Kpn*I and ligated to the plasmid pUC18 to generate plasmid pMW833. A *aph*(*3*′)-*III* cassette obtained from plasmid vector pBHP489K was inserted into plasmid pMW833 digested with *Afe*I. This plasmid was designated as plasmid pMW834 and transformed into J99 and SW861 (*flaB* mutant). Km (10 μg ml^−1^) was used to select for the *flaA* mutant (SW866) and *flaA*/*flaB* double mutant (SW868).

### Construction of the *flaB* promoter mutants and revertant

The primers used for construction of the *flaB* promoter mutants and revertant are listed in Table S1. The 1,001 bp *flaB* promoter fragment obtained from *H. pylori* J99 genomic DNA and PCR with flaB-ProMut-1 and flaB-ProMut-2 primers, was digested by *Hinc*II and *EcoR*V and ligated to the plasmid pUC18 to generate plasmid pMW814. Primers flaB-ProMut-3 and flaB-ProMut-4 were used to amplify pMW814 to generate plasmid pMW813 containing a 46 bp partial deletion of the *flaB* promoter and carrying a *Nae*I site in the *flaB* promoter fragment. A *cat* cassette obtained from plasmid vector 78 was inserted into plasmid pMW813 digested with *Nae*I. This plasmid was designated as plasmid pMW812 and transformed into J99. Cm was used to select for the *flaB* promoter-*cat* mutant, SW856. In order to construct *flaB* promoter mutants with GG or CG/GG nucleotide mutations, primer pairs flaB-ProMut-7/flaB-ProMut-8 and flaB-ProMut-9/flaB-ProMut-10 were used to amplify pMW814 to generate plasmid pMW811 and pMW810, respectively. pMW814, pMW813, pMW811, and pMW810 were transformed into SW856 to generate SW857 (revertant), SW855 (deletion), SW853 (CG to AA/GG to AA), and SW854 (GG to AA), respectively.

### Soft-agar motility assay

The motility assay was described previously (Kao et al., 2014). Bacterial colonies were applied to one spot in the motility agar plate containing Brucella broth, 0.3% (w/v) Bacto agar, and supplemented with 10% (v/v) horse serum. The plates were incubated at 37°C under microaerophilic conditions for 7 days, and the motility was assessed by the diameter of migration of bacteria through the agar, from the inoculated center toward the periphery of the plate. The motility of each strain was measured as the mean of three independent experiments.

### Reconstruction of three-dimensional model of Jhp0106 protein

Complete amino acid sequence of *H. pylori* J99 Jhp0106 (Accession number: WP_001028953) was downloaded from NCBI database (http://www.ncbi.nlm.nih.gov/protein/). A three-dimensional structural modeling was carried out on the SWISS-MODEL Workspace server (http://swissmodel.expasy.org/) (Arnold et al., 2006). The structure representation figures are generated by the program PyMOL (http://www.pymol.org).

### Transmission electron microscope

A grid covered with a carbon-coated parlodion film (300 mesh copper grid) was floated onto a 20 μl sample drop and left for 2 min for adsorption of the sample to the grid. The grid was then removed from the drop and floated on a drop of 1% (w/v) phosphotungstic acid (Sigma-Aldrich) and left for 1 min. Excess stain was removed by touching the edge of the grid to a piece of Whatman filter paper. All samples for electron microscopy were examined in a Hitachi H-7650 transmission electron microscope (Hitachi, Tokyo, Japan).

### Bacterial adhesion and IL-8 production assay

The assay was performed according to a previous study with modification (Kwok et al., 2002). AGS and GES-1 cells (1 × 10^6^/well) were grown overnight in 6-well culture dishes to approximately 80% confluence. *H. pylori* cells were added to the wells at a multiplicity of infection (MOI) of 100 without centrifugation and were incubated for either 30 min for the adhesion assay or 8 h for the cellular IL-8 production assay. For the adhesion assay, each dish with AGS-*H. pylori* coculture was washed three times with prewarmed phosphate-buffered saline (PBS) buffer to remove unbound bacteria. Adhered *H. pylori* were quantified by lysing the cells for 15 min with 0.1% (w/v) saponin-containing PBS buffer, followed by serial dilution and spreading on Brucella agar plates containing 10% (v/v) horse serum. The number of adhered bacteria was measured by the plate counts after 3 days incubation. For the cellular IL-8 production assay, the culture supernatants were collected and stored at −20°C until assayed. IL-8 concentration in the supernatant was determined by standard ELISA with commercially available assay kits according to the manufacturer's procedures (Arigo Biolaboratories Corp., Taiwan).

### Statistics

The Student's *t-*test and paired *t*-tests were applied as appropriate for the parametric differences. ANOVA was used for comparing groups of more than two strains. All tests of significance were two-tailed with a *p* value < 0.05 taken as significant.

## Results

### CsrA acts as a global positive regulator in strain J99

To characterize the CsrA regulatory system, RNA-seq analysis on the wild-type J99 and its respective *csrA* mutant, SW835, was carried out. Three independent biological replicates were sequenced for each strain. The raw sequence output of the two strain transcriptomes included ~14 and ~15 million reads of the wild-type and *csrA* mutant, respectively. Approximately 80% of the reads were perfectly aligned to the J99 reference genome. Based on the genomic alignment, our analysis determined the expression of 1,559 genes in each strain.

In this study, genes found to be differentially expressed compared to the wild-type J99 (a > 1.5-fold change) by RNA-seq data were taken into consideration. In the *csrA* mutant, 53 chromosomally encoded genes were found to be differentially expressed compared to the wild-type J99 (Table 2). Of these genes, 94% (50 genes) were expressed at a lower level in the *csrA* mutant compared to the wild-type, with only 6% (3 genes) having higher expression in the *csrA* mutant (Table 2). These results indicate that CsrA acts primarily as a global positive regulator. RT-qPCR was further employed to validate the expression of 53 CsrA regulated genes in the wild-type and *csrA* mutant (Table 2). In agreement with the RNA-seq data, RT-qPCR confirmed the expression of genes differentially expressed in the *csrA* mutant compared to the wild-type J99 with a > 2-fold change (except *jhp1296*). Three genes, *jhp1334, jhp1169* and *jhp1132*, originally identified with a >1.5-fold change by RNA-seq, showed inconsistent results between the RNA-seq and RT-qPCR methods (change <1.5-fold by RT-qPCR) (Table 2). These transcriptomic analyses also indicated that most of CsrA regulated genes (18 genes) were classified as genes encoding components involved in flagella formation, chemotaxis and motility, followed by genes with unknown function (15 genes) (Figure S1).

**Table 2:** **Gene ID, annotation and function of genes regulated by the CsrA regulatory system identified by RNA-seq analysis**.

**Gene ID (J99)**	**Gene ID (26695)**	**Annotation**	**Fold change (RNA-Seq/RTqPCR)^a^**
**METABOLISM**			
jhp0099 (*cysK*)	HP0107	Cysteine synthase	−2.05^**^/−2.27
jhp0158 (*moeA*)	HP0172	Molybdopterin biosynthesis protein	−1.57^**^/−1.62
jhp0338 (*ribF*)	HP1087	Riboflavin kinase	−3.44^***^/−2.39
jhp0528 (*pyrC_2*)	HP0581	Dihydroorotase	−3.57^***^/−2.98
jhp1334 (*ppiA*)	HP1441	Peptidyl-prolyl cis-trans isomerase	−1.80^***^/−1.09
**NUCLEOTIDE METABOLISM**			
jhp0549	HP0602	3-methyladenine DNA glycosylase	−1.61^*^/−2.05
jhp0691	HP0754	5-formyltetrahydrofolate cyclo-ligase	−4.19^***^/−4.97
jhp1050	HP1121	DNA methyltransferase	−3.80^***^/−2.95
jhp1296	–^b^	Type II restriction endonuclease	−2.56^**^/−1.27
**CHEMOTAXIS AND MOTILITY**			
jhp0106	HP0114	Unclear	−3.70^**^/−4.37
jhp0107 (*flaB*)	HP0115	Flagellin B	−3.99^***^/−3.04
jhp0280 (*flgL*)	HP0295	Flagellar hook-associated protein 3 (HAP3)	−3.66^**^/−4.20
jhp0349	HP1076	Cochaperone	−8.13^***^/−6.26
jhp0374	HP1051	Unclear	−2.74^***^/−2.20
jhp0548 (*flaA*)	HP0601	Flagellin A	−2.94^***^/−2.73
jhp0688 (*flaG*)	HP0751	Putative flagellin protein	−4.38^***^/−3.32
jhp0689 (*fliD*)	HP0752	Putative flagellar hook-associated protein 2 (HAP2)	−4.76^***^/−4.09
jhp0690 (*fliS*)	HP0753	Putative flagellar protein	−5.12^***^/−5.23
jhp0751 (*motA*)	HP0815	Flagellar motor protein	−1.53^**^/−3.07
jhp0752 (*motB*)	HP0816	Flagellar motor protein	−1.67^***^/−1.93
jhp0804 (*flgE*)	HP0870	Flagellar hook protein	−1.52^*^/−7.32
jhp0842	HP0906	FliK functional homolog	−3.24^**^/−2.50
jhp1047 (*flgK*)	HP1119	Flagellar hook-associated protein 1 (HAP1)	−4.88^***^/−3.28
jhp1048	HP1120	Unclear	−4.95^***^/−3.96
jhp1051 (*flgM*)	HP1122	Sigma-28 factor antagonist	−3.24^***^/−3.14
jhp1154	HP1233	Unclear	−1.83^*^/−1.66
jhp1169	HP1248	Ribonuclease II family protein	−1.61^***^/−1.46
**REGULATOR**			
jhp0652 (*rpoN*)	HP0714	RNA polymerase sigma-54 factor	−3.09^***^/−3.18
**TRANSPORT**			
jhp0167	HP0179	ABC transporter, ATP-binding protein	−1.60^**^/−5.59
jhp0396	HP1028	Lipocalin family protein	−2.06^**^/−2.28
jhp0529 (*tonB*)	HP0582	Siderophore-mediated iron transport protein	−3.22^***^/−3.24
jhp0653	HP0715	ABC transporter, ATP-binding protein	−3.22^***^/−3.72
jhp0743 (*fecA*)	HP0807	Iron (III) dicitrate transport protein	−1.75^ns^/−3.37
**CELL SURFACE STRUCTURE**			
jhp0373 (*lpxC*)	HP1052	UDP-3-O-hydroxymyristoyl	−2.72^***^/−2.31
jhp0424	HP0472	Outer membrane protein (omp11)	−5.06^***^/−4.49
jhp0526	HP0579	Kdo hydrolase subunit 2	−4.01^**^/−2.64
jhp0527	HP0580	Kdo hydrolase subunit 2	−3.03^***^/−2.04
**STRESS RESPONSE**			
jhp0423 (*kefB*)	HP0471	Glutathione-regulated potassium efflux system protein	−2.23^***^/−3.00
**PUTATIVE OR UNKNOWN FUNCTION**			
jhp0436	HP0484	Putative	−3.38^***^/−2.45
jhp0550	HP0603	Putative	−2.17^***^/−2.24
jhp0572	HP0629	Putative	1.54^*^/3.54
jhp0753	HP0817	Putative	−1.54^***^/−1.69
jhp0936	–^b^	Putative	−1.78^***^/−3.24
jhp1049	–^b^	Putative	−3.53^***^/−2.94
jhp1242	HP1322	Putative	2.60^**^/2.48
jhp1302	HP1409	Putative	2.83^ns^/2.70
jhp1332	HP1439	Putative	−1.85^ns^/−1.23
jhp1333	HP1440	Putative	−7.22^***^/−11.63
jhp1430	HP1397	Putative	−3.23^***^/−2.97
jhp1431	HP1396	Putative	−3.74^***^/−4.12
jhp1436	HP1391	Putative	−2.16^**^/−2.13
jhp1437	–^b^	HcpA family protein	−1.63^**^/−1.69
jhp1474	HP1566	Membrane protein	−1.77^***^/−1.88

a *Gene expression in the csrA mutant compare to wild-type J99 with a change > 1.5-fold is listed in this table. ns, not significant*;

* *p < 0.05*;

** *p < 0.01*;

*** *p < 0.001*.

b *Strain-specific genes in H. pylori J99*.

### CsrA/RpoN regulates *flaB*-*jhp0106* expression

The CsrA-regulated *jhp0106* gene was of interest, as it encodes a protein suggested to be involved in *H. pylori* strain 1016 flagellin glycosylation and motility (Schirm et al., 2003) (Table 3). The schematic diagram showing the pseudaminic acid biosynthetic pathway in *H. pylori* is shown in Figure S2. At present, the function of Jhp0106 in flagellin glycosylation is still unclear. We validated the transcription of *pseB, pseC, pseH, pseG, pseI* and *jhp0106* in the wild-type, SW835 (*csrA* mutant) and SW836 (*csrA* revertant) strains by RT-qPCR. The results were consistent with the RNA-seq data. Only the expression of *jhp0106* was dramatically reduced in SW835 compared to the wild-type J99 (Figure S3).

**Table 3:** **Proteins showing high similarity to Jhp0106**.

**Bacteria**	**Protein**	**Function**	**a.a (no.)**	**Identities to Jhp0106 (%)**	**References**
*Calditerrivibrio nitroreducens*	Calni_0724	Unclear	633	30	–
*Campylobacter jejuni*	Cj1340c	Motility accessory factor	605	30	Golden and Acheson, 2002
*Campylobacter jejuni*	Maf1	Motility accessory factor	649	35	Karlyshev et al., 2002
*Campylobacter jejuni*	Maf3	Motility accessory factor	619	34	McNally et al., 2006
*Campylobacter jejuni*	Maf4	Flagellin glycosylation	649	35	van Alphen et al., 2008
*Campylobacter jejuni*	Maf6	Motility accessory factor	607	29	Karlyshev et al., 2002
*Campylobacter jejuni*	PseD	Flagellin glycosylation	653	36	McNally et al., 2006
*Campylobacter jejuni*	PseE	Flagellin glycosylation	628	34	McNally et al., 2006
*Caminibacter mediatlanticus*	CMTB2_07872	Unclear	631	41	–
*Denitrovibrio acetiphilus*	Dacet_0453	Unclear	633	29	–
*Helicobacter mustelae*	HMU07160	Unclear	629	62	–
*Helicobacter pylori*	Jhp0106	Motility accessory factor	627	100	Schirm et al., 2003
*Nautilia profundicola*	NAMH_1610	Motility accessory factor	643	44	–
*Sulfurospirillum deleyianum*	Sdel_2228	Unclear	627	42	–
*Syntrophomonas wolfei*	Swol_0199	Unclear	671	28	–

**Figure 1:**
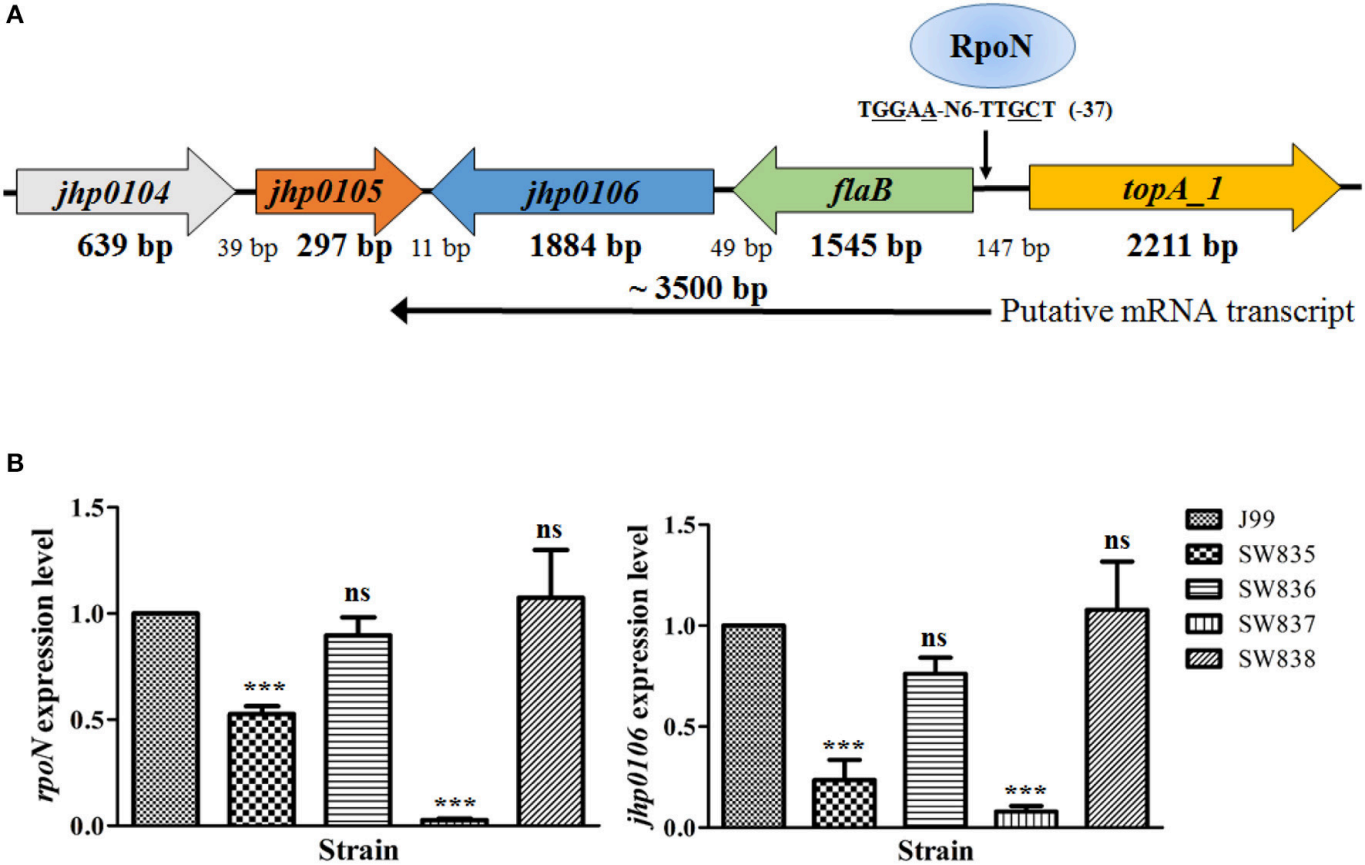
**Reduced expression of *jhp0106* in strains SW835 and SW837. (A)** Schematic diagram showing the genetic loci of genes close to the putative *flaB*-*jhp0106* operon. A predicted RpoN binding site located upstream of the *flaB* ORF (−37 bp) is indicated. **(B)** RT-qPCR was used for quantifying the mRNA levels of *rpoN* (left panel) and *jhp0106* (right panel) in examined strains. Results are representative of 3 independent experiments (means ± SD). ^***^p < 0.001, ns, not significant (vs. wild-type J99). SW835, *csrA* mutant; SW836, *csrA* revertant; SW837, *rpoN* mutant; SW838, *rpoN* revertant.

The genes close to *jhp0106* in *H. pylori* J99 are shown in Figure 1A. *jhp0106* was located immediately downstream of the *flaB* gene, which has been shown to be regulated by RpoN. The gene order and orientation of *flaB*-*jhp0106* are conserved in *H. pylori* genomes. Therefore, we propose genes *flaB* and *jhp0106* are in an operon controlled by RpoN binding to the *flaB* promoter. To validate this hypothesis, RT-qPCR was carried out to determine the expression of *rpoN* and *jhp0106* in the wild-type, SW835 (*csrA* mutant), SW836 (*csrA* revertant), SW837 (*rpoN* mutant), and SW838 (*rpoN* revertant) strains. The results showed that the expression of *rpoN* was reduced in SW835 (Figure 1B), consistent with our previous study (Kao et al., 2014). Moreover, expression of *jhp0106* mRNA was decreased to 24 and 8% in SW835 and SW837, respectively (*p* < 0.001). These results indicated that CsrA and RpoN positively regulate *jhp0106* expression.

**Figure 2:**
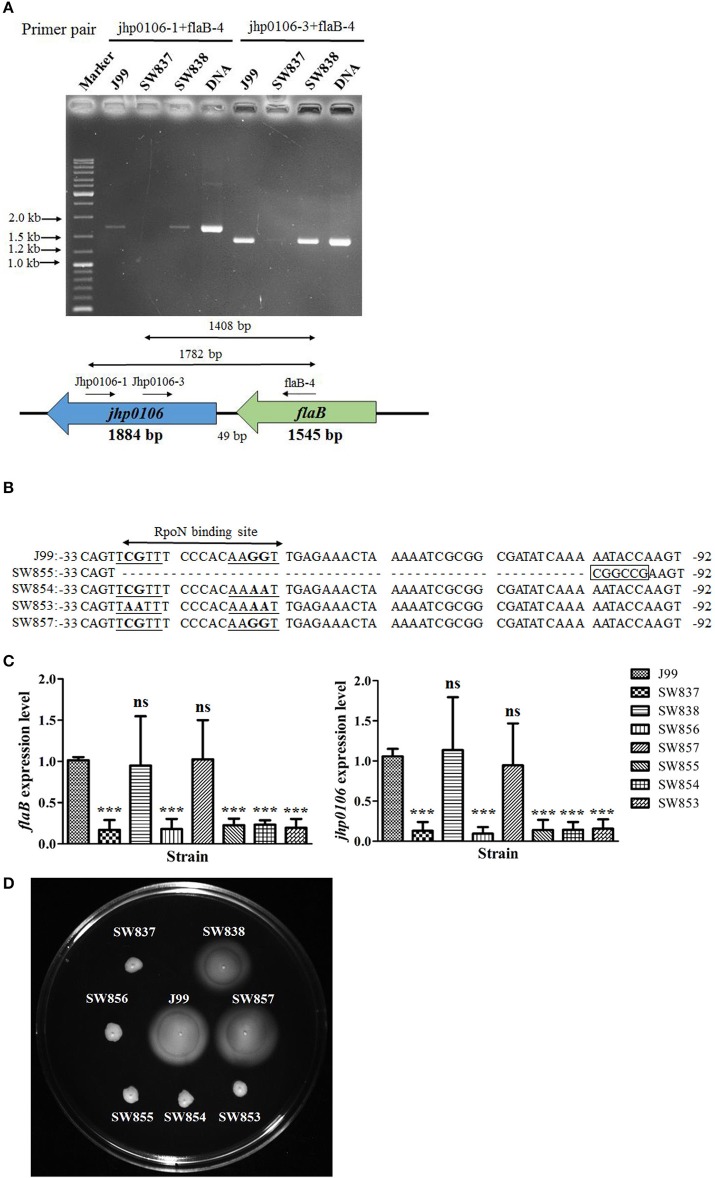
**Characterization of the *flaB*-*jhp0106* operon. (A)** The expression of the *flaB*-*jhp0106* co-transcript in J99, SW837, and SW838 was determine by RT-PCR. The primer pairs and predicted PCR product size are described in the lower panel. J99 DNA was considered as a positive control. Marker, GeneRuler™ DNA ladder (Fermentas). **(B)** Sequence alignment of the *flaB* promoter region (−33 to −92 bp, upstream of the *flaB* ORF) of wild-type, SW855, SW854, SW853, and SW857 strains. **(C)** mRNA levels of *flaB* and *jhp0106* in 8 examined strains were measured by RT-qPCR. Results are representative of 3 independent experiments (means ± SD). ^***^p < 0.001, ns, not significant (vs. wild-type J99). **(D)** The motility of 8 tested strains was determined by soft-agar motility assay plates. SW837 was used as a negative control (non-motile phenotype). SW837, *rpoN* mutant; SW838, *rpoN* revertant.

RT-PCR with different primer pairs was performed to confirm the co-transcription of *flaB*-*jhp0106*, and the results showed that 1.4- and 1.8-Kb transcripts were observed using primer pairs jhp0106-1/flaB-4 and jhp0106-3/flaB-4 for RT-PCR, respectively (Figure 2A). In addition, the expression of the *flaB*-*jhp0106* transcript was reduced in SW837 (Figure 2A). The predicted RpoN binding site of the *flaB* promoter was examined by construction of *flaB* promoter mutants, as shown in Figure 2B. The mRNA levels of *flaB* and *jhp0106* were dramatically reduced in SW837, SW853, SW854, SW855, and SW856, as determined by RT-qPCR (Figure 2C). We further investigated whether the RpoN binding site of the *flaB* promoter was conserved among different *H. pylori* strains, and the results showed that the RpoN binding sequence was identical among 17 examined strains (Figure S4). Our results indicated the transcription of *jhp0106* is positively controlled by RpoN bound to the *flaB* promoter. However, these results could not exclude the possibility that the *jhp0106* gene had its own promoter. Therefore, northern blotting was performed to determine the size and number of *jhp0106* transcripts. Although several different fragments of the *jhp0106* gene were used to serve as probes, the signal was still too weak to be detectable (data not shown).

In order to clarify the role of the *flaB*-*jhp0106* operon in *H. pylori* J99 motility, soft-agar analysis was used to determine the motility of *flaB* promoter mutants (Figure 2D). SW837 was used as a non-motile negative control. The results showed that strains SW853, SW854, SW855, and SW856 exhibited deficient motility (Figure 2D). These results indicated that the *flaB*-*jhp0106* operon was controlled by RpoN, and that it plays a critical role in *H. pylori* motility.

### Jhp0106, but not flaB, plays a critical role in *H. pylori* motility

**Figure 3:**
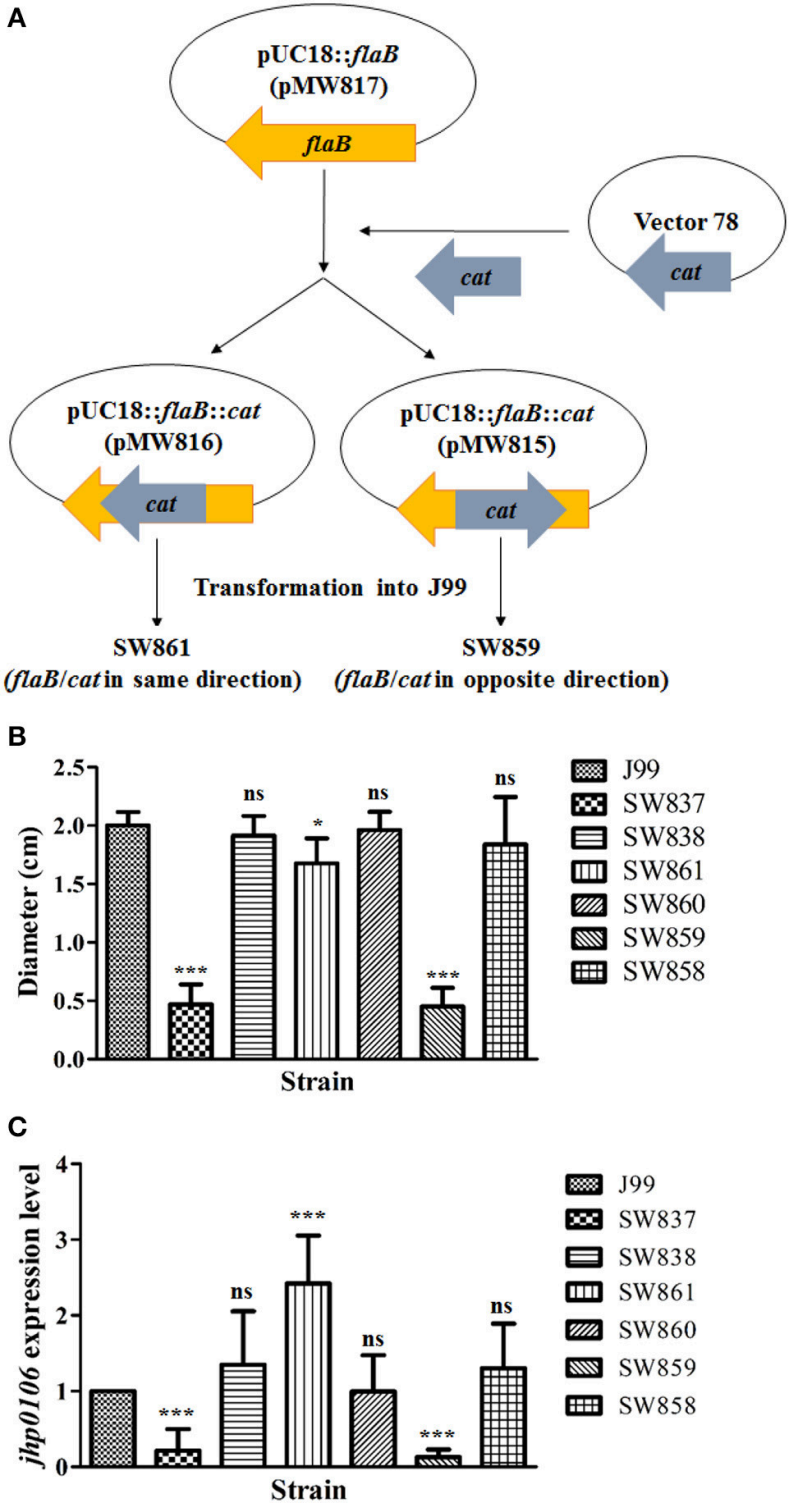
**Characterization of the influence of *flaB* and *jhp0106* on *H. pylori* motility. (A)** Schematic diagram showing the construction strategy of the *flaB* mutants, SW861 and SW859. **(B)** The quantified motility diameter of 7 examined strains. SW837 was used as a negative control (non-motile phenotype). **(C)** The mRNA level of *jhp0106* was determined by RT-qPCR. Results are representative of 3 independent experiments (means ± SD). ^*^p < 0.05, ^***^p < 0.001, ns, not significant (vs. wild-type J99). SW837, *rpoN* mutant; SW838, *rpoN* revertant.

Josenhans et al. reported that when the *flaB* gene was disrupted in *H. pylori* strain N6, the motility decreased by 30 to 40% (Josenhans et al., 1995). To validate this observation in strain J99, we constructed *flaB* mutants SW861 and SW859 (Figure 3A). A *cat* cassette was inserted in the same (SW861) or opposite (SW859) direction to *flaB* transcription in wild-type J99 (Figure 3A). Strain SW861 showed decreased motility, and the quantified results revealed 83.8% motility ability compared with wild-type J99 (*p* < 0.05) (Figure 3B). In contrast, strain SW859 showed a non-motile phenotype, compared with the wild-type J99 (Figure 3B). To evaluate whether the deficient phenotype of SW859 was caused by the insertion of the *cat* cassette into the *flaB* gene, which interfered with downstream *jhp0106* expression, RT-qPCR was performed to determine the expression of *jhp0106* in the examined strains. The results showed that the transcription level of *jhp0106* was decreased to 13% in SW859, but increased to 242% in SW861, compared to the wild-type (Figure 3C). The increase of *jhp0106* expression in SW861 resulted from a *cat* cassette inserted into *flaB* in same transcriptional orientation. Thus triggering downstream *jhp0106* transcription due to the leakage of the transcriptional terminator of the *cat* cassette. These results indicated that *jhp0106* in *flaB*-*jhp0106* operon, but not *flaB*, plays a critical role in *H. pylori* motility.

### Jhp0106, a putative glycosyltransferase

The NCBI protein database was used to search for proteins showing high similarity to Jhp0106, and the results are shown in Table 3. Among them, HMU07160 (62%) showed the highest identity to Jhp0106, followed by WS2199 (55%), NAMH_1610 (44%), Sdel_2228 (42%), and CMTB2_07872 (41%) (Table 3). The phylogenic tree based on the homologous full-length sequence of 16 proteins was also displayed in Figure S5. Previous studies reported that Maf4, PseD and PseE were involved in flagellin glycosylation in *C. jejuni* (McNally et al., 2006; van Alphen et al., 2008), and they also displayed high identity to Jhp0106 (Table 3).

**Figure 4:**
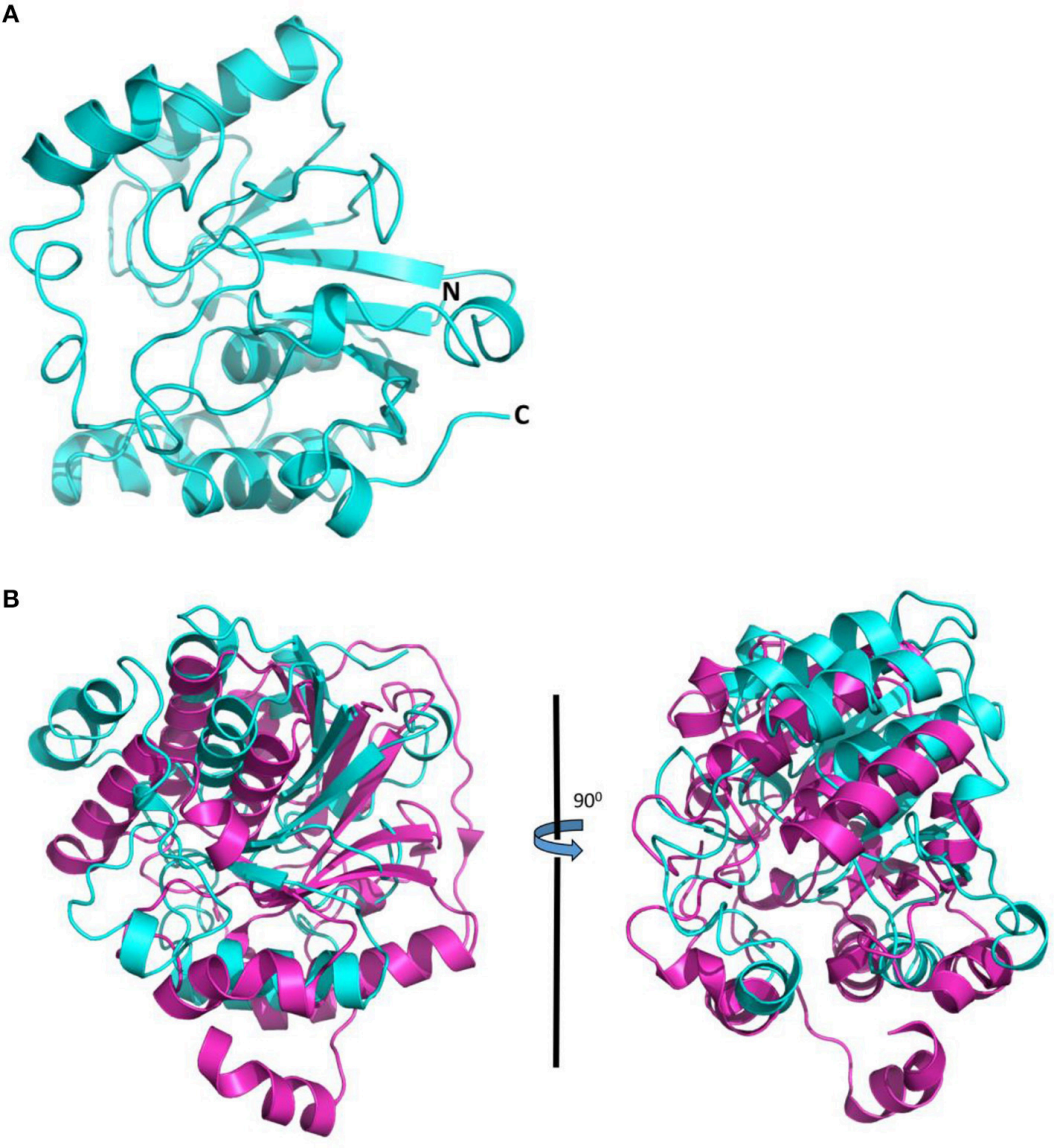
**Computational structure model of Jhp0106 from *H. pylori* J99. (A)** The structural model of Jhp0106 composed of 227 amino acids, by the SWISS-MODEL server. **(B)** Structural superimposition of Jhp0106 (cyan) with the crystal structure of sialyltransferase CstII in complex with CMP-3FNeuAc (PDB 1R07) (magenta).

The attempt to determine the crystal structure of Jhp0106 was unsuccessful, in spite of extensive efforts to crystallize the recombinant Jhp0106 protein from an *E. coli* expression system. Therefore, we performed computational modeling of the Jhp0106 protein to gain structural insights into Jhp0106 function. A 3D-model for Jhp0106 of 227 amino acid residues in length (from Asp231 to Phe457) predicted by the SWISS-MODEL server is shown in Figure 4A. The overall folding of the Jhp0106 structure is similar to an alpha-2,3/8-sialyltransferase CstII from *C. jejuni* in complex with a substrate analog, CMP-3FNeuAc (PDB: 1R07) (Figure 4B) (Chiu et al., 2004), suggesting Jhp0106 is a glycotransferase.

The protein interaction networks for Jhp0106 showed that the protein interacts with flagella structure proteins (FlaA, FlaB, FlaG, FlaL, FliD), pseudaminic acid synthase (NeuB), CagDelta protein (Jhp0417), hypothetical proteins (PdP, Jhp0578), and a septum formation inhibitor (MinC) (Figure S6). These results suggested that Jhp0106 functions as a glycosyltransferase and is involved in the transfer of pseudaminic acid to flagellin.

### Jhp0106 is involved in flagella formation

The roles of Jhp0106 in *H. pylori* flagella formation, adhesion and cellular cytokine induction were examined by constructing *jhp0106* mutant (SW863) and revertant J99 (SW862) strains. The genetic loci of *jhp0105* and *flaB* are close to *jhp0106* in the J99 genome (Figure 1A). In order to rule out the possibility of polar effects in SW862 and SW863, the expression of *jhp0105, jhp0106* and *flaB* were determined by RT-PCR (Figure S7). The data revealed that the mRNA expression levels of *jhp0105* and *flaB* in strains SW863 and SW862 were similar to J99, and the transcription of *jhp0106* was only disrupted in SW863 (Figure S7). In addition, the 72 h growth curves of SW863 and SW862 were similar to that of the wild-type J99 (Figure S8).

**Figure 5:**
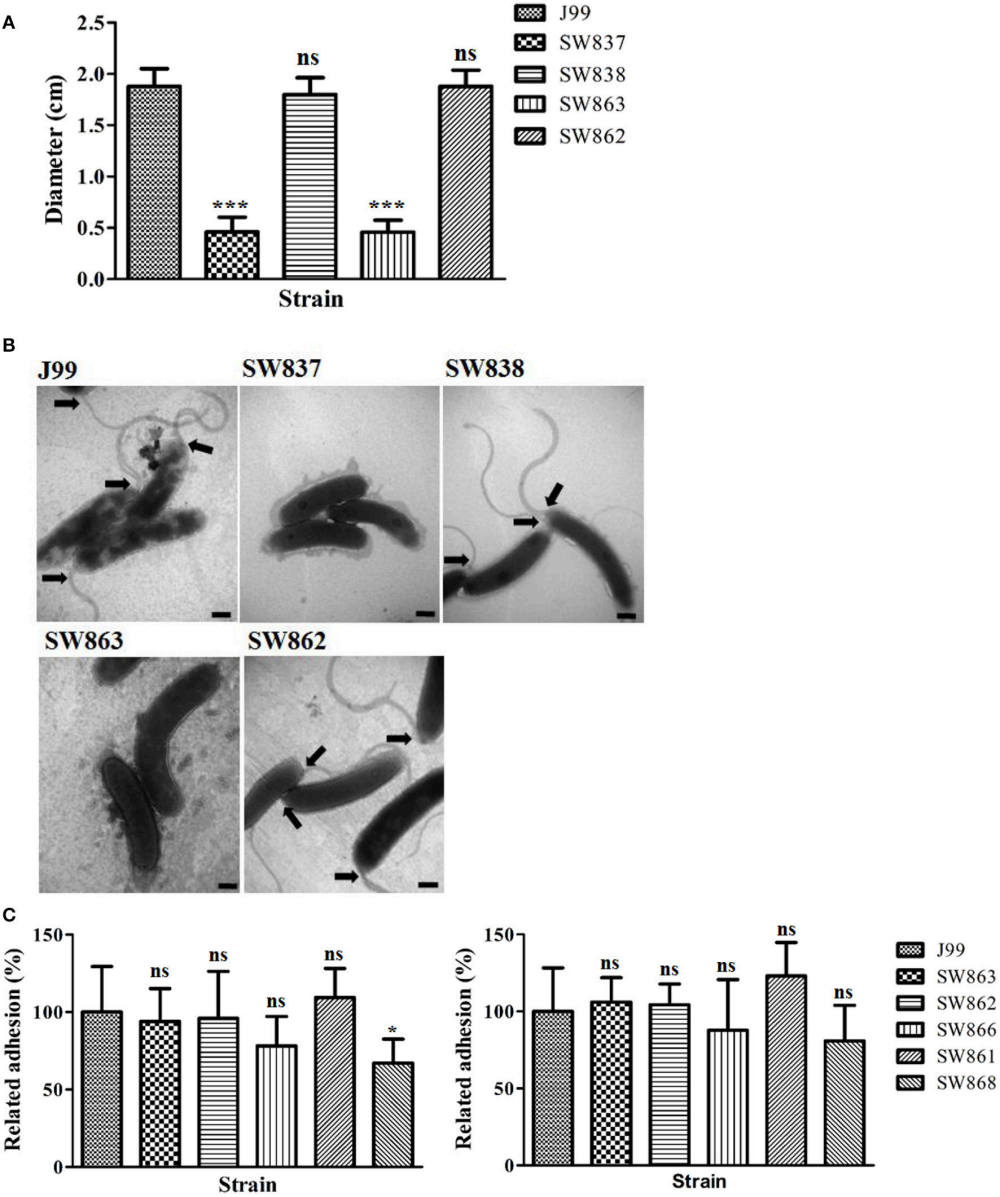
**Motility, flagellar structure, and adhesion ability of strains J99, SW837, SW838, SW863, and SW862. (A)** The quantified motility diameter of tested strains. SW837 was used as a negative control (non-motile phenotype). **(B)** Transmission electron micrographs of negatively stained *H. pylori*. Black arrowheads indicate the presence of full-length flagella at the cell poles. Scale bars represent 500 nm. **(C)** The adhesion of *H. pylori* to AGS cells (left panel) and GES-1 cells (right panel) with MOI 100 of the tested strains. Results are representative of 3 independent experiments (means ± SD). ^*^p < 0.05, ^***^p < 0.001, ns, not significant (vs. wild-type J99). SW837, *rpoN* mutant; SW838, *rpoN* revertant; SW863, *jhp0106* mutant; SW862, *jhp0106* revertant; SW866, *flaA* mutant; SW861, *flaB* mutant; SW868, *flaA*/*flaB* mutant.

The deficient motility of SW863 is shown in Figure 5A. The bacterial shape and flagellar structure of J99, SW837, SW838, SW862, and SW863 were examined by TEM with negative staining. No flagellar structure was detected in SW837 and SW863, whereas the characteristic multiple polar, sheathed flagella were abundant on J99, SW838 and SW862 (Figure 5B). In addition, there was no dramatic difference in shape between the strains examined. These observations demonstrated that flagella formation was severely defective in the *jhp0106* mutant.

To determine the role of flagella structure and Jhp0106 in *H. pylori* pathogenesis, the adhesion rate of the bacteria and the IL-8 production in *H. pylori*-infected AGS and GES-1 cells were determined (Figure 5C and Figure S9). The results showed that only SW868 (*flaA*/*flaB* double mutant) had lower adhesion to AGS cells, compared with J99 (*p* < 0.05) (Figure 5C). No difference in IL-8 production was observed between cells infected with the six examined strains, as determined by an ELISA assay (*p* > 0.05) (Figure S9).

### Characterization of Jhp0106 in clinical isolates

**Figure 6:**
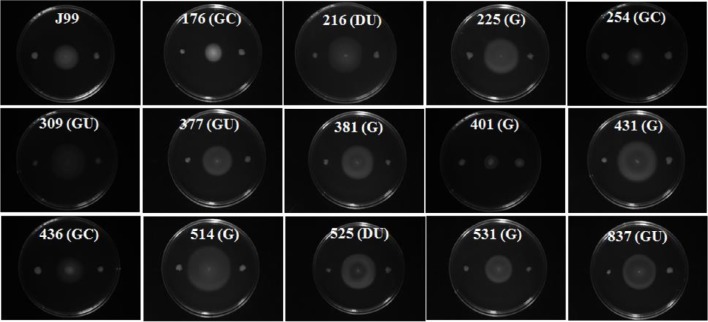
***rpoN*** **and *jhp0106* are critical for the motility of 15 *H. pylori* strains**. Wild-type, *rpoN* mutant, and *jhp0106* mutant strains were inoculated in the middle, left, and right of the soft-agar plate, respectively. G, gastritis; GU, gastric ulcer; DU, duodenal ulcer; GC, gastric cancer.

Many studies suggest that genetic diversity in *H. pylori* virulence factors such as *sabA, babA, cagA*, and *vacA* genes is high among isolates from different geographic regions, and may be associated with different pathological outcomes (van Doorn et al., 1998; Yamaoka et al., 2006). As a result, we evaluated the prevalence of the *jhp0106* gene in 95 isolates from patients with different diseases by PCR, including 38 gastritis strains, 21 duodenal ulcer strains, 17 gastric ulcer strains, 18 gastric cancer strains, and 1 MALToma strain. The results showed that all tested isolates contained the *jhp0106* gene. To determine whether Jhp0106 is a critical factor in *H. pylori* motility among different strains, we constructed *rpoN* and *jhp0106* mutants of 14 clinical isolates. The results of soft-agar motility assay showed that all *rpoN* mutants and *jhp0106* mutants were non-motile (Figure 6).

## Discussion

The present study aimed to reveal the CsrA regulatory system by using RNA-seq, and to identify CsrA target genes involved in *H. pylori* motility. We showed that CsrA regulated the level of the *flaB*-*jhp0106* transcript in J99 by controlling the expression of the alternative sigma factor RpoN. In addition, Jhp0106 was characterized as a putative glycosyltransferase involved in flagellin glycosylation and flagella formation.

Edwards et al. identified > 700 transcripts that bind to CsrA in *E. coli*, indicating that CsrA affects expression of ~15% of the genes in *E. coli* (Edwards et al., 2011). In this study, we revealed that 53 genes (~4%) in a *csrA* mutant were found to be differentially expressed, compared to the wild-type J99 (Table 2). Like CsrA in *E. coli* and *Clostridium acetobutylicum* (Edwards et al., 2011; Tan et al., 2015), RNA-seq analysis showed that CsrA in *H. pylori* was closely involved in regulating multiple pathways including metabolism, iron uptake, flagella assembly, and oligopeptide transport (Table 2). However, the molecular mechanism through which CsrA regulates target gene expression in strain J99 remained to be clarified. Moreover, 15 CsrA-regulated genes of unknown function are worth investigating further (Table 2).

CsrA has been shown to control target gene expression by diverse mechanisms in several organisms (Romeo et al., 2013). For example, CsrA is mainly known for its post-transcriptional role in mRNA stability (Liu et al., 1995; Wei et al., 2001; Baker et al., 2002; Wang et al., 2005; Esquerre et al., 2016). In other cases, CsrA binds to multiple sites in the untranslated leader and/or initially translated region of target transcripts, and bound CsrA thus repressed translation by competing with ribosome binding to the Shine-Dalgarno sequence, but did not affect the level of targeted mRNA (Dubey et al., 2003; Ren et al., 2014). This may explain why only 4% of genes in J99 were identified to be regulated by CsrA, while 15% of genes were CsrA-regulated in *E. coli* (Edwards et al., 2011). As a result, it is worth carrying out a comparative proteomic analysis to evaluate any CsrA post-transcriptionally regulated genes in *H. pylori*.

Barnard et al. showed that the morphology of the *csrA* mutant N6 strain was similar to the wild-type strain, with a unipolar bundle of four or five flagella (Barnard et al., 2004). mRNA levels of *flaA* and *flaB* were elevated in the *csrA* mutant compared to the N6 strain. In addition, mutation of *csrA* in the N6 strain resulted in the increased expression of neutrophil activating protein (*napA*), ferric uptake regulator (*fur*), *hspR*, and *groESL* (Barnard et al., 2004). However, this is in contrast to our results. The expression of *flaA* and *flaB* at the mRNA level was reduced in the *csrA* mutant J99 (Table 2). *napA, fur, hspR*, and *groESL* transcripts were not changed in the *csrA* mutant J99. These results raised the possibility that the CsrA regulatory system was strain-specific, due to the high variation of the *H. pylori* genome.

Douillard et al. indicated that the *hp0256* mutant has lower motility, significantly weaker adhesion, and induces weaker IL-8 secretion in AGS cells compared to the wild-type CCUG17874 strain (Douillard et al., 2010). In our previous study, we showed that in patients infected with higher-motility strains, the bacterial density, inflammatory score, and rate of atrophy were higher than those of patients infected with lower motility strains (Kao et al., 2012a), suggesting that *H. pylori* motility may be positively correlated with bacterial adhesion and *il-8* gene expression in *H. pylori*-infected AGS cells. However, the *jhp0106* mutation caused a non-flagellated phenotype in strain J99, and there was no decrease of adhesion or IL-8 production in AGS and GES-1 cells infected with SW863, compared to the wild-type (Figure 5, Figure S9). In contrast, the *flaA*/*flaB* double mutant (SW868), showing deficient motility, had weaker adhesion to AGS cells (67% compared to the wild-type) (Figure 5C). These results indicated that the motility of J99 has a minor role in cell adhesion. However, whether Jhp0106 modulates the composition/or modification of surface proteins in J99 and thus affects adhesion to AGS-1 cells is still unclear.

Based on the computational modeling, the overall structure of Jhp0106 resembles CstII, which is reported to participate in the sialylation of lipooligosaccharide cores and thus affects the immunogenicity of *C. jejuni* (Guerry et al., 2000; Chiu et al., 2004). Ram et al. showed that the sialylation of gonococcal lipo-oligosaccharide enables *Neisseria gonorrhoeae* to bind the alternative pathway complement inhibitor, factor H, and thus provides a protective barrier to evade attack by human complement (Ram et al., 1998). However, the role of pseudaminic acid in bacterial pathogenesis remains unclear. Pseudaminic acid has been shown to have striking structural and biosynthetic similarities to sialic acid (Lewis et al., 2009). Taken together, we suggested that Jhp0106 is involved in the transfer of pseudaminic acid to flagellin FlaA/FlaB, but not in the pseudaminic acid biosynthetic pathway (Figure S2). Therefore, large-scale identification of Jhp0106 target proteins by an LC/MS-based glycoproteomic approach is worth investigating.

The current treatments for *H. pylori* eradication are numerous and include triple and quadruple therapy, both of which utilize two antibiotics (metronidazole, amoxicillin, tetracycline, or clarithromycin) in addition to either a proton pump inhibitor (PPI) (triple therapy), or a PPI and bismuth (quadruple therapy) (O'Connor et al., 2016). The efficacies of these therapy strategies have been severely hampered in recent years due to the rise in antibiotic resistance of *H. pylori* isolates worldwide. Therefore, there is an emergent need to develop alternative therapeutic strategies for the management of *H. pylori* infection. Currently, carbohydrate-based therapies and diagnostics in cancer research and infectious disease have received considerable attention. Menard et al. identified three inhibitors of the pseudaminic acid biosynthetic enzymes which show activity in inhibiting the flagellin proteins on the *C. jejuni* cell surface, by bacterial cell-based assays (Menard et al., 2014). In this study, we found the prevalence of the *jhp0106* gene among 95 clinical isolates of *H. pylori* in Taiwan was 100%. Moreover, mutation of *jhp0106* of 15 clinical strains (include J99) led to the loss of motility of all mutants (Figure 6). These results suggest that Jhp0106 is a promising target for developing an inhibitor to restrain *H. pylori* infection in the future.

**Figure 7:**
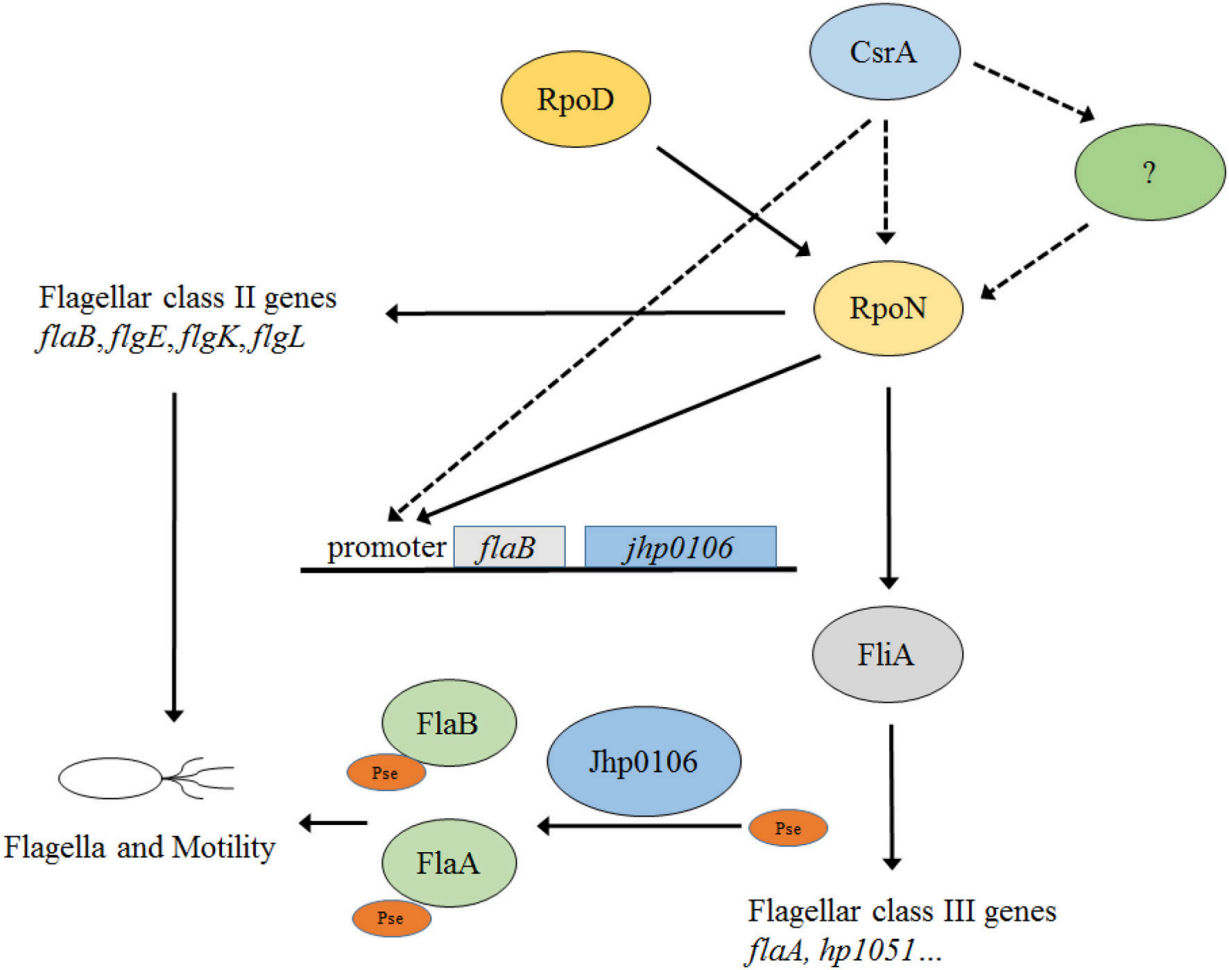
**Current model of the CsrA/RpoN flagellar biosynthesis and motility regulatory system in *H. pylori* J99**. Three different classes of flagellar genes are governed by the housekeeping sigma factor RpoD (class I), and the alternative sigma factors RpoN (class II) and FliA (class III). CsrA positively controls *H. pylori* J99 flagella formation and motility through regulating *rpoN* expression by an unclear mechanism(s) (shown in dotted line). In this study, the expression of *jhp0106* (with a putative glycosyltransferase function) is under the control of the CsrA/RpoN system through the binding of RpoN to *flaB* promoter.

CsrA controls flagella-related genes' expression and motility of J99 by regulating RpoN expression (Kao et al., 2014) (Figure 7). Although RpoN is the key regulator under the control of CsrA, the mechanism(s) through which CsrA modulates *rpoN* expression is still unclear (Figure 7). In this study, we reveal the CsrA regulatory system in *H. pylori* by large-scale identification of target genes using RNA-seq. Moreover, the results suggest that RpoN not only controls flagellin expression but also modulates flagella assembly by regulating the expression of the putative glycosyltransferase Jhp0106, and thus affects the post-translational modification of flagellin (Figure 7). The motility of *H. pylori* is a critical virulence determinant in bacterial pathogenesis, therefore, understanding the complex regulatory pathways of flagella formation could in the future lead to novel therapies against *H. pylori* colonization. Future work will focus on the characterization of the Jhp0106 protein, including localization, enzymatic residues, and target proteins.

## Author contributions

CK, JC, and SW conceived the study, carried out experimental work and drafted the manuscript. CK, BS, and JW helped to interpret the data and draft the manuscript. All authors have read and approved the final manuscript.

### Conflict of interest statement

The authors declare that the research was conducted in the absence of any commercial or financial relationships that could be construed as a potential conflict of interest. The reviewer TC and handling Editor declared their shared affiliation, and the handling Editor states that the process nevertheless met the standards of a fair and objective review.
