# Supplementary material for: The Helicobacter pylori J99 jhp0106 Gene, under the Control of the CsrA/RpoN Regulatory System, Modulates Flagella Formation and Motility
Source: Front Microbiol. 2017 Mar 28;8:483. doi: 10.3389/fmicb.2017.00483 (PMC5368276; doi:10.3389/fmicb.2017.00483)
Supplement: Supplementary file 1 [file Table1.doc]

**Experimental procedures**

***flaB promoter sequence alignment***

To compare the RpoN binding sequence of the *flaB* promoter among different *H. pylori* strains, 17 *flaB* promoter sequences from the NCBI database (http://www.ncbi.nlm.nih.gov/nuccore/) were downloaded. Multiple nucleotide sequence alignment was performed by importing the corresponding nucleotide sequences into the CLC Workbench.

***Phylogenetic tree analysis***

To draw a phylogenetic tree of the Jhp0106 homologous proteins, 16 protein sequences from the NCBI database (http://www.ncbi.nlm.nih.gov/protein/) were downloaded (Accession number: HMU07160, CBG39973; WS2199, CAE11189; NAMH_1610, WP_012663848; Sdel_2228, ACZ13240; CMTB2_07872, WP_007474653; PseD, CAL35446; PseE, CAL35449; Maf3, CAL35447; Maf4, CAL35448; Maf1, CAL35432; Dacet_0453; WP_013009795; Cj1340c, CAL35452; Maf6, CAL35453; Swol_0199, WP_011639660; Calni_0724, ADR18635). All 16 sequences were first aligned using the CLC Workbench (CLC sequence viewer 7.0, CLC Bio/Qiagen, Aarhus, Denmark), and the aligned file was then subjected to unweighted pair group method with arithmetic mean (UPGMA) to draw a phylogenetic tree with a bootstrap value of 50.

***Protein interaction network analysis***

Interactions of Jhp0106 were evaluated by STRING (Search Tool for the Retrieval of Interacting Genes, http://string-db.org/) (Snel *et al*., 2000), which is the online database resource that provides both experimental and predicted interaction information (Szklarczyk *et al*., 2015).

**References**

1. Snel, B., Lehmann, G., Bork, P., and Huynen, M.A. (2000). STRING: a web-server to retrieve and display the repeatedly occurring neighbourhood of a gene. Nucleic Acids Res. 28, 3442–3444.
2. Szklarczyk, D., Franceschini, A., Wyder, S., Forslund, K., Heller, D., Huerta-Cepas, J., [Simonovic, M](https://www.ncbi.nlm.nih.gov/pubmed/?term=Simonovic M%5BAuthor%5D&cauthor=true&cauthor_uid=25352553)., et al. (2015). STRING v10: protein-protein interaction networks, integrated over the tree of life. Nucleic Acids Res. 43, D447–452.

**Table S1. Oligonucleotide primers used in this study.**

| Primer | Sequence (5’-3’) |
| --- | --- |
| **Mutant construction** |  |
| flaB-ProMut-1 | ATGATATCGACGCTTTAACCGTGGTGTT |
| flaB-ProMut-2 | ATGATATCTAAAATCGCATTTTGCGTGA |
| flaB-ProMut-3 | ACTCTTTGATTTTTAGCGCCGCTATAG |
| flaB-ProMut-4 | AGCCGGCTGACTAACAGCAAATATCTATGCAAAGG |
| flaB-ProMut-7 | GATATTTGCTGTTAGTCAATTAAAGGGTGTTCCAACTCTTTG |
| flaB-ProMut-8 | GGCGCTAAAAATCAAAGAGTTAAAACACCCTTTGCTTGACTA |
| flaB-ProMut-9 | GATATTTGCTGTTAGTCAATTAAAGGGTGTTTTAACTCTTTG |
| flaB-ProMut-10 | GGCGCTAAAAATCAAAGAGTTAAAACACCCT |
| flaB-Mut-1 | AACTGCAGCATATCCGAGCGGATCTTGT |
| flaB-Mut-2 | GGGGTACCTAAGGAGTCAAAGCGCGAAT |
| jhp0106-Mut-1 | CCGGAATTCCGCTTCTTCAACTTGCGTTT |
| jhp0106-Mut-2 | CCGGAATTCAAATCAACCAGCATTTCACG |
| FlaA-Mut-1 | AACTGCAGCATGGGGATTATCCAGGTTG |
| FlaA-Mut-2 | GGGGTACCTGGCTTGACTCATCGCATAG |
| **RT-PCR** |  |
| jhp0106-1 | GCTGTTACCGCTTTGTGAAA |
| jhp0106-13 | AATTTCGCCGTTAAATCCAC |
| flaB-4 | AGGACCGACGCTAGAGACAT |
| jhp0105-1 | TGACGCAACCATAAAGGACA |
| jhp0105-2 | TCTTTAATCTCGCCGTCGTT |
| **RT-qPCR** |  |
| gyrA-RealT-1 | GACACCGCAGTTTATGATGC |
| gyrA-RealT-2 | TTCTGGCTTCAGTGTAACGC |
| jhp0099(cysK)-qPCR-1 | AAGTGCCACCACTCCCTATC |
| jhp0099(cysK)-qPCR-2 | TATTTGCCCTTGCAATTTGA |
| jhp0158(moeA)-qPCR-1 | TCTTTAGGAGCTAGGGCGAA |
| jhp0158(moeA)-qPCR-2 | GGTGCCAAAGGGAATAGAAA |
| jhp0338(ribF)-qPCR-1 | ATGCCCAAACCTGAAATCAT |
| jhp0338(ribF)-qPCR-2 | GCCAAACTCGTTAAGATGCC |
| jhp0528(pyrC_2)-qPCR-1 | CGCATAGAGGTTGTCATGCT |
| jhp0528(pyrC_2)-qPCR-2 | AAACTTTCGCCCTTTCATTC |
| jhp1334(ppiA)-qPCR-1 | GGGCTTAACTTCCATCGTGT |
| jhp1334(ppiA)-qPCR-2 | TTATGAGCCACTTCGCATTT |
| jhp0248-qPCR-1 | GAATAAGCATGGCAAGCAAA |
| jhp0248-qPCR-2 | CGGTGCGTCTTGAGATATTG |
| jhp0549-qPCR-1 | CCAAACGACTGATTGATTTGA |
| jhp0549-qPCR-2 | GGCGCACACATAGCATAAA |
| jhp0691-qPCR-1 | GCTTGGATTTAGATGCGCTTT |
| jhp0691-qPCR-2 | AGCCTTATGCATTTGCGATT |
| jhp1050-qPCR-1 | CCGGTTATGGTTGTATGGGT |
| jhp1050-qPCR-2 | GAAACGCCCATTTAATTTCC |
| jhp1296(mod)-qPCR-1 | CTGTTCGTTATCGTCGCATT |
| jhp1296(mod)-qPCR-2 | CCACAGCTCTTGGCTAGTGT |
| jhp0106-qPCR-1 | CTTCAGGCAAATTAGCGACA |
| jhp0106-qPCR-2 | AAATCAACCAGCATTTCACG |
| jhp0107(flaB)-qPCR-1 | ATTCACGGTGTATTCTGCCA |
| jhp0107(flaB)-qPCR-2 | AGGACCGACGCTAGAGACAT |
| jhp0280(flgL)-qPCR-1 | AATGAATCGCTCCTTGCTTT |
| jhp0280(flgL)-qPCR-2 | TCAAGCGTCTAAACCCAATG |
| jhp0349-qPCR-1 | AGCTTGCAAAGCGTCTTTATC |
| jhp0349-qPCR-2 | CAAGTCTTGCAGATCGCTTG |
| jhp0374-qPCR-1 | GCTTAAAGCTCACGCATGTT |
| jhp0374-qPCR-2 | AAATCAAAGCCTGTGGTTGA |
| jhp0548(flaA)-qPCR-1 | AATGGCGGTCAGGATTTAAC |
| jhp0548(flaA)-qPCR-2 | TGTGAGTCAGAAGCCGAAAC |
| jhp0688(flaG)-qPCR-1 | CGAGCGTTTGAATGAAGAAA |
| jhp0688(flaG)-qPCR-2 | AGCGTCTTTGACTGAAACCA |
| jhp0689(fliD)-qPCR-1 | GAGCATGCATGAAGTCCCTA |
| jhp0689(fliD)-qPCR-2 | CTAAAGCCTGCTCCATAGCC |
| jhp0690(fliS)-qPCR-1 | TTTAACGCAAGCCAATGTG |
| jhp0690(fliS)-qPCR-2 | CCTCCATGCCTCTAACAACC |
| jhp0804(flgE)-qPCR-1 | AAACATGCCGTATTGGTTGA |
| jhp0804(flgE)-qPCR-2 | ATTGGGCAGTTTAGGACCAC |
| jhp0842-qPCR-1 | CGAGCGATCATCTTGCTTTA |
| jhp0842-qPCR-2 | GCGTCGTTCTTTGTAAGGGT |
| jhp1047(flgK)-qPCR-1 | GATTGTCATCGGTGTTAGCG |
| jhp1047(flgK)-qPCR-2 | ACCGATGGTAAAGAAATCGC |
| jhp1048-qPCR-1 | AAAGACAACATTTGCGCATC |
| jhp1048-qPCR-2 | CGAGCGCGATATAAGAGACA |
| jhp1051(flgM)-qPCR-1 | TCAATCGCTTGCTTGATTTC |
| jhp1051(flgM)-qPCR-2 | TCACTCCAGTGCAATCTGTG |
| jhp1154-qPCR-1 | TCCGTCAAGCTATAAAGCGA |
| jhp1154-qPCR-2 | ACCCTGACAAGCGAGAGACT |
| jhp1169(vacB)-qPCR-1 | GATCGATCTTTACCACGCAA |
| jhp1169(vacB)-qPCR-2 | CCAGATTGTGGCTATCCCTT |
| jhp0652(rpoN)-qPCR-1 | AAGCCCTCTTTCACGTCTTT |
| jhp0652(rpoN)-qPCR-2 | CGAAGTTTATGAGAAAGTACGCA |
| jhp0167-qPCR-1 | TTGATTAGCGCTAGTGGTGTC |
| jhp0167-qPCR-2 | CATCGCCAGAGTGCTTTCTA |
| jhp0396-qPCR-1 | TTTCTGATGTGGATGGCTCT |
| jhp0396-qPCR-2 | TTATACGCCTTACCGCCTTT |
| jhp0529(tonB_1)-qPCR-1 | TCGGTAATATCGCCGTTAGG |
| jhp0529(tonB_1)-qPCR-2 | TAGCGGCTTATTTAGGGCAA |
| jhp0653-qPCR-1 | ACACGCCAATGTTTAATCCA |
| jhp0653-qPCR-2 | TCGTGCTGTTAGATGAGCCT |
| jhp0743(fecA_2)-qPCR-1 | GCATGCCGTTATTGAAATTG |
| jhp0743(fecA_2)-qPCR-2 | GATTTATACCGCCGATCCAC |
| jhp0751(motA)-qPCR-1 | TGAAGACGATTTCACCCGTA |
| jhp0751(motA)-qPCR-2 | CGGCACCGTGGTAATACTC |
| jhp0752(motB)-qPCR-1 | TCAGCGGTCAATAAATCCAA |
| jhp0752(motB)-qPCR-2 | CGCCATTTGCTCTTCTTCTT |
| jhp0373(lpxC)-qPCR-1 | TTGAGGTTAGAGAGGGCGAT |
| jhp0373(lpxC)-qPCR-2 | CGACTTGCTCTTTGTAAGCG |
| jhp0424-qPCR-1 | CTAATTGAACGCCACCAATG |
| jhp0424-qPCR-2 | CAGGGACTGAACACACCAAA |
| jhp0526-qPCR-1 | AAAAAGCGCGCTAAAAATCA |
| jhp0526-qPCR-2 | AGCCCCCTTTAGAAACCTTG |
| jhp0527-qPCR-1 | TGCGAATGATGAGAAAGCTC |
| jhp0527-qPCR-2 | TTAGCGGCACTAAAGAAGGG |
| jhp0423(kefB)-qPCR-1 | TGGCTGCAATATCTTGGAAA |
| jhp0423(kefB)-qPCR-2 | CGCCATTGTGTTGAAATTCT |
| jhp0436-qPCR-1 | GTTCAAAGCGGATTTCAAGG |
| jhp0436-qPCR-2 | TGCACCACAATAGTGGGAAT |
| jhp0550-qPCR-1 | ATGGGCGATTCGTATTTCAT |
| jhp0550-qPCR-2 | CACCGCTGATTGGATATTGA |
| jhp0572-qPCR-1 | GGTTTAGAGTTGGGCGAAGA |
| jhp0572-qPCR-2 | TTTAATTGCACGCCTGTAGC |
| jhp0753-qPCR-1 | GAATTGAAAGAGCAGGAGCA |
| jhp0753-qPCR-2 | TAAATAGGGCGTTTCTTGGG |
| jhp0936-qPCR-1 | CATTGACACTATGGCGGAGTT |
| jhp0936-qPCR-2 | AAAATGCCCTTAAGCCTTTCA |
| jhp1049-qPCR-1 | TCTGCCGTTATCACCTCCTT |
| jhp1049-qPCR-2 | CATGAAATGGGCGTTAAAAA |
| jhp1242-qPCR-1 | TGTGCAATCGCATTGTTAAA |
| jhp1242-qPCR-2 | GCACCATGACATACACACGA |
| jhp1302-qPCR-1 | AAATGGTTACGCCCTGTCTT |
| jhp1302-qPCR-2 | CGTTCCATTGACTGCCTCT |
| jhp1332-qPCR-1 | CGAAAGACAGAGCAAGAATCC |
| jhp1332-qPCR-2 | AGGGATAAGACACCCACAGC |
| jhp1333-qPCR-1 | CTTGATCCCTATCCATCGCT |
| jhp1333-qPCR-2 | GGGCGTTATATCGCTTCTTC |
| jhp1430-qPCR-1 | TTCCGAAACTAACGCTGAAA |
| jhp1430-qPCR-2 | TTTGCCAGCAGAATGAAAGT |
| jhp1431-qPCR-1 | CAGCAAATTGTATGAACAACACA |
| jhp1431-qPCR-2 | ACATGCAAATCGTCATCCAT |
| jhp1436-qPCR-1 | TGCTGGATTTAGCACAAAGC |
| jhp1436-qPCR-2 | TTATCGCTAATGGCTTGCAT |
| jhp1437-qPCR-1 | AAAGCTTGCAAATCAAACGA |
| jhp1437-qPCR-2 | TTCAATGCTCTCTCTTGCGT |
| jhp1474-qPCR-1 | TGCATGCTTTCAAACCAAAA |
| jhp1474-qPCR-2 | CGACCATTTTCTGCCAAGTT |
| PseB-RealT-1 | ATTAACCTCTACGGCGCAAC |
| PseB-RealT-2 | CCCACCACATTACCATAACG |
| PseC-RealT-1 | TTCTTTCTGACAGCTCGCAT |
| PseC-RealT-2 | GCCGTGGTGATAGGCTTAAT |
| PseH-RealT-1 | TTACCCAGTTAAACGATGAAGAAA |
| PseH-RealT-2 | CTTCTATGAATTGCAAATGCG |
| PseG-RealT-1 | TGAATTTGCACATCATTTCATC |
| PseG-RealT-2 | GCGCAAGATTTCATCAAAGA |
| PseI-RealT-1 | AAAGTGGTGGCTAGAATGGG |
| PseI-RealT-2 | CTTTGGCGTTAAATTTGGCT |

**Figures**

**Fig. S1**

**
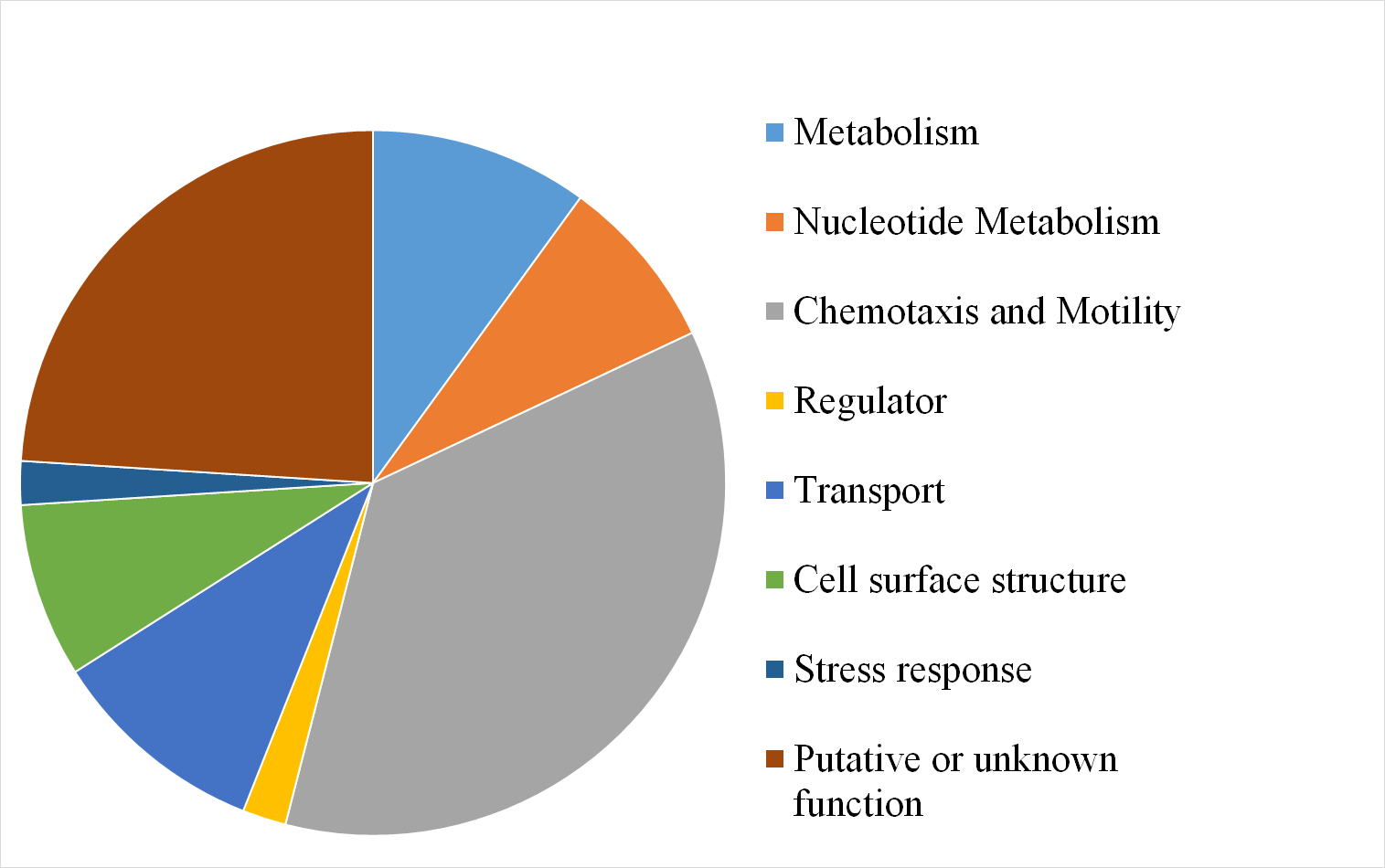
**

**Fig. S1.** Classification of fifty genes downregulated in the *csrA* mutant, by gene ontology. The expression of 3 genes with unknown function was upregulated in the *csrA* mutant.

**Fig. S2**

**
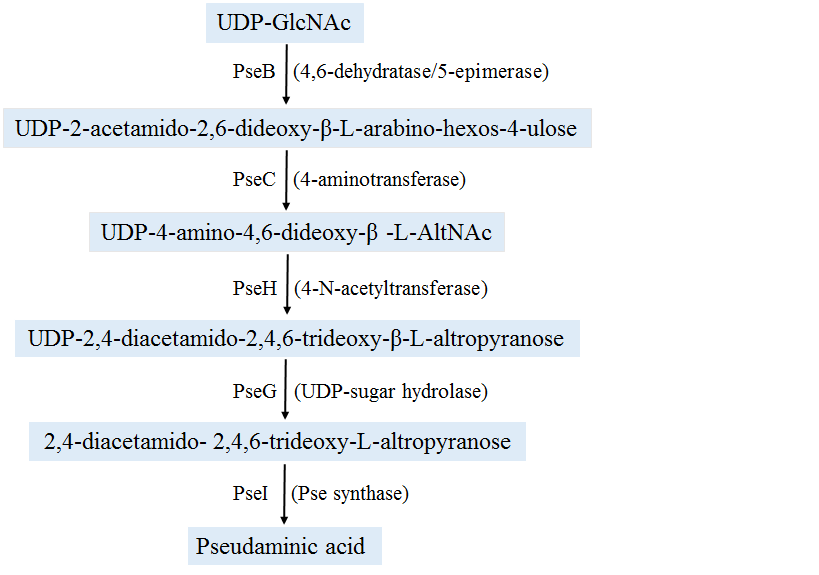
**

**Fig. S2.** Schematic diagram showing the pseudaminic acid biosynthetic pathway in *H. pylori*.

**Fig. S3**

**
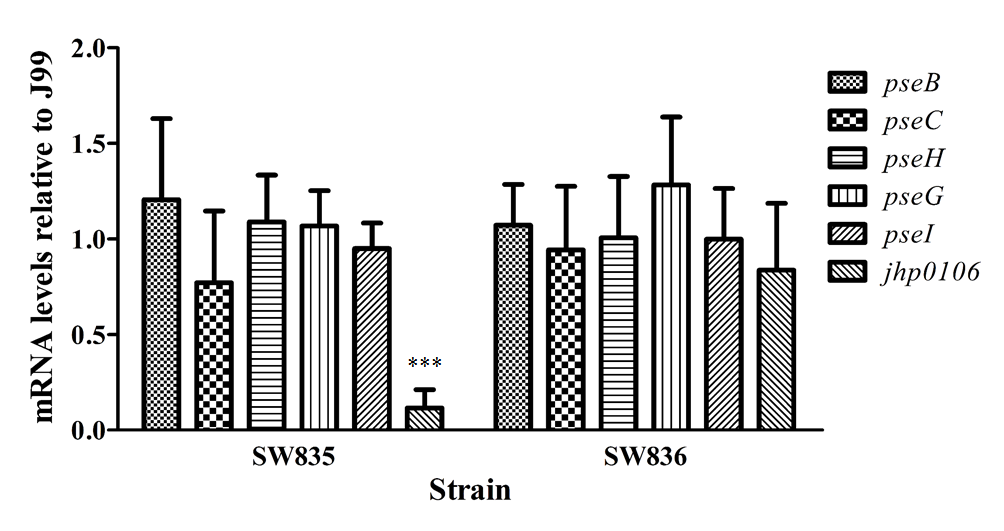
**

**Fig. S3.** mRNA levels for the pseudaminic acid biosynthetic enzymes and *jhp0106* of J99, SW835 and SW836 were measured by RT-qPCR. Results are representative of 3 independent experiments (means ± SD). *** = *p* < 0.001 (vs. wild-type J99). SW835, *csrA* mutant; SW836, *csrA* revertant.

**Fig. S4**

**
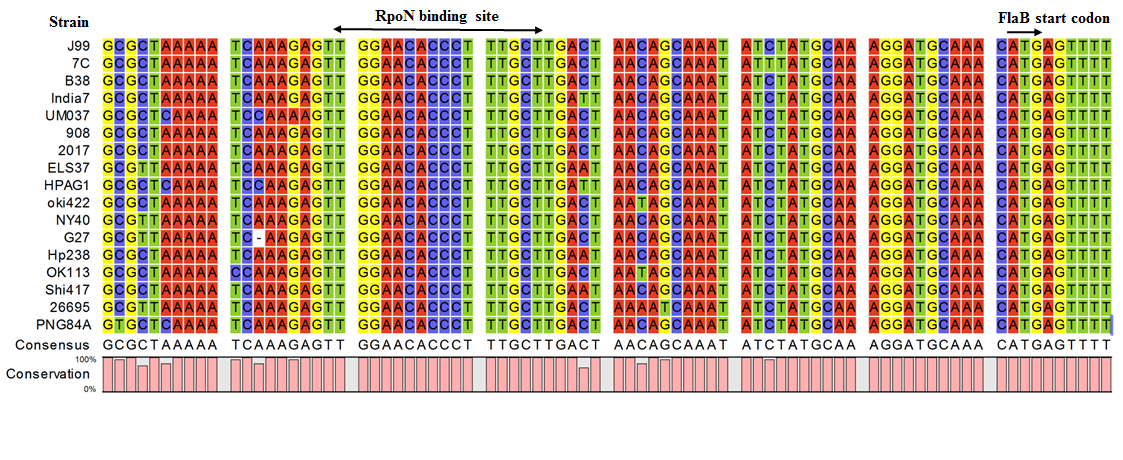
**

**Fig. S4.** Sequence alignment of the *flaB* promoter region of 17 *H. pylori* strains, using the CLC sequence viewer.

**Fig. S5**

**
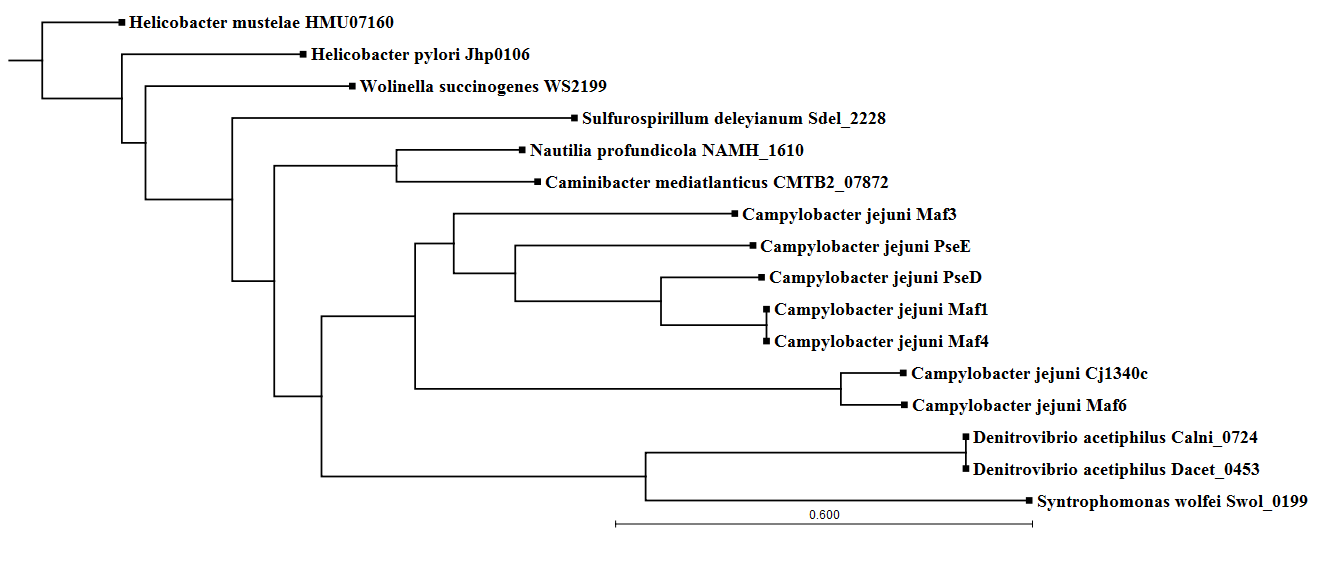
**

**Fig. S5.** A phylogenetic tree of species based on protein sequences, constructed using the UPGMA method. The lengths of the nodes represent the substitution rate, which is defined as the percentage of substitution sites per alignment length. Fifty bootstrap replications were performed using the CLC sequence viewer 7.0 software.

**Fig. S6**

**
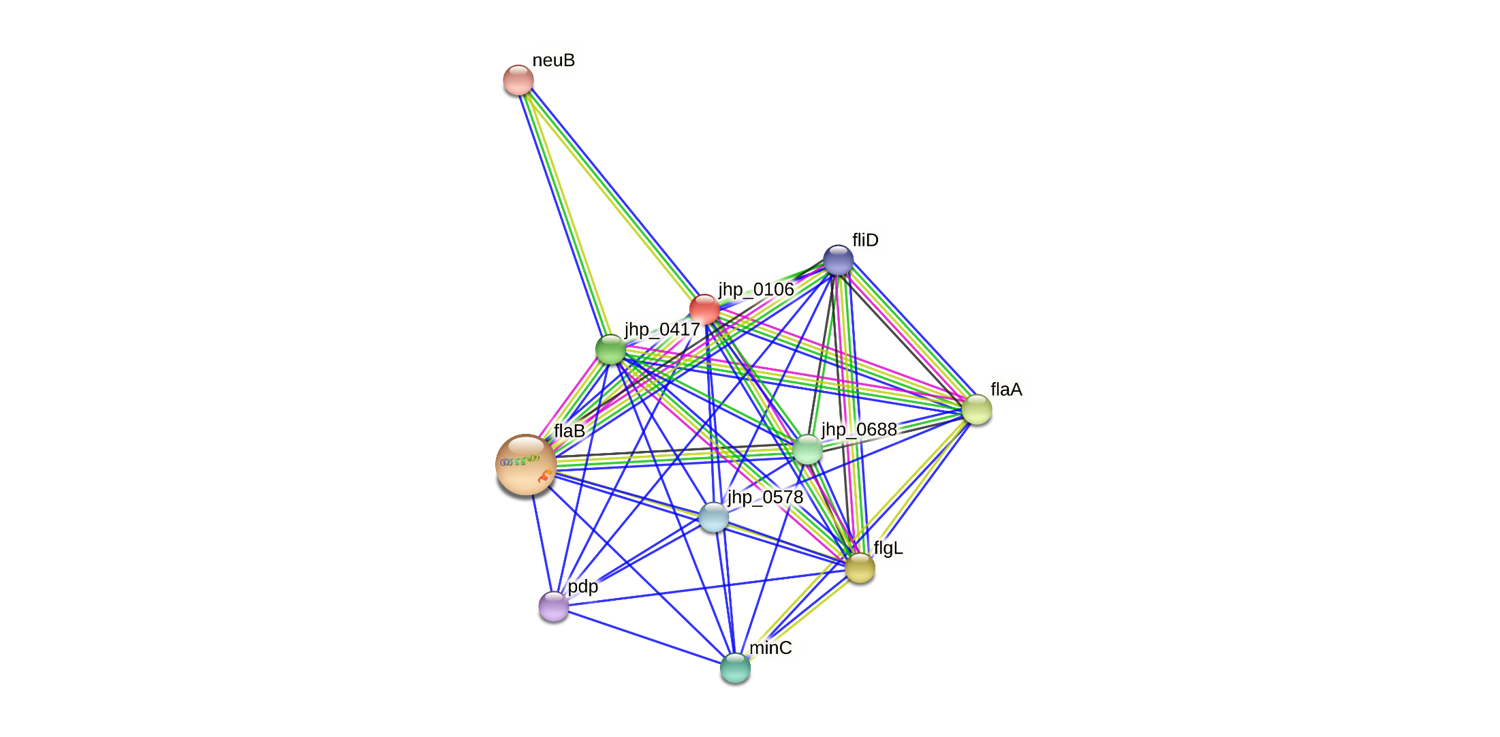
**

**Fig. S6.** STRING produced the network of predicted associations for a particular group of proteins related to Jhp0106. The predicted functional partners are: *flaB* (flagellin B); *flgL* (flagellar hook-associated protein); *flaA* (flagellin A); *jhp_0417* (CagDelta protein); *jhp0688* (flagellar protein FlaG); *minC* (septum formation inhibitor); *jhp0578* (hypothetical protein); *fliD* (flagellar capping protein); *pdp* (hypothetical protein); and *neuB* (pseudaminic acid synthase). Colored lines denote interactions: green (neighborhood), blue (co-occurrence) purple (experiments), and light green (textmining).

**Fig. S7**

**
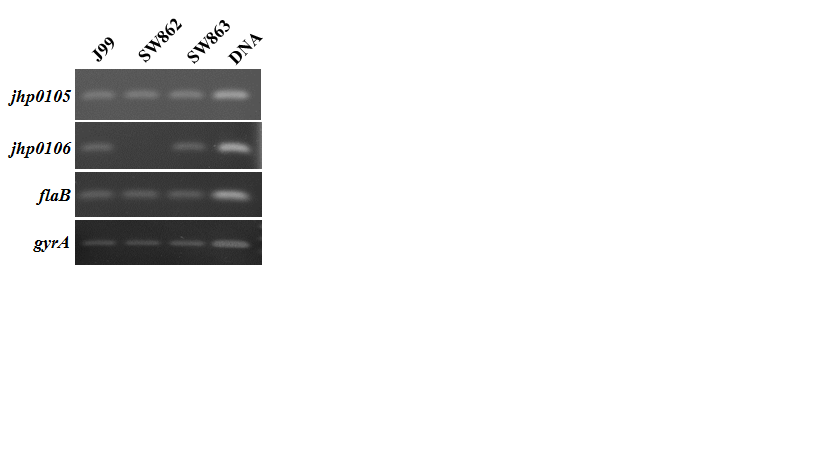
**

**Fig. S7.** The expression of *jhp0105*, *jhp0106* and *flaB* in J99, SW863 (*jhp0106* mutant) and SW862 (*jhp0106* revertant) was determined by RT-PCR. The expression of *gyrA* was used as the internal control.

**Fig. S8**

**
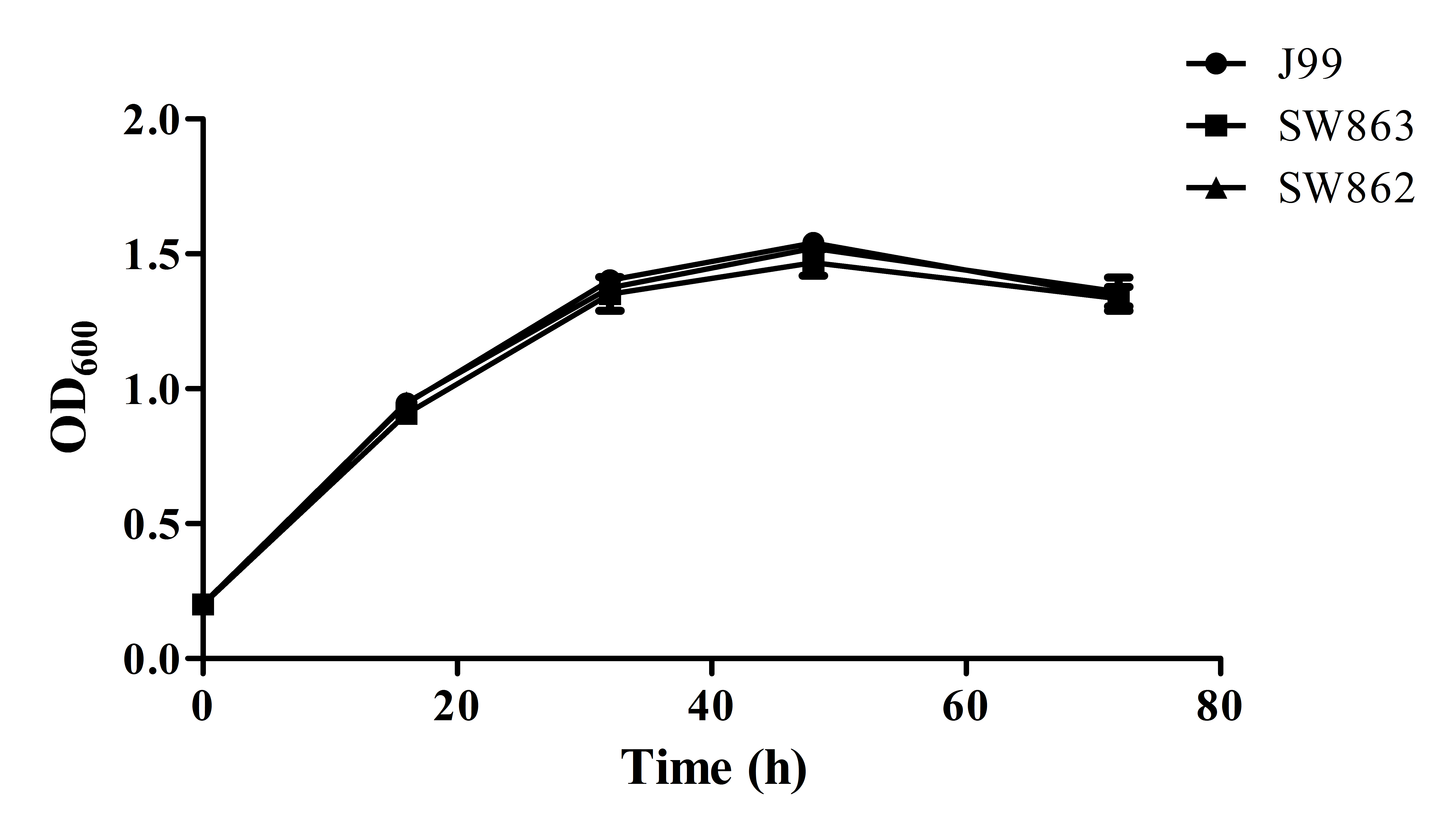
**

**Fig. S8.** The growth of J99, SW862 (*jhp0106* mutant) and SW863 (*jhp0106* revertant) (as measured by culture density) was determined at different time points, and the results showed that the growth curves of the strains were similar.

**Fig. S9**

**
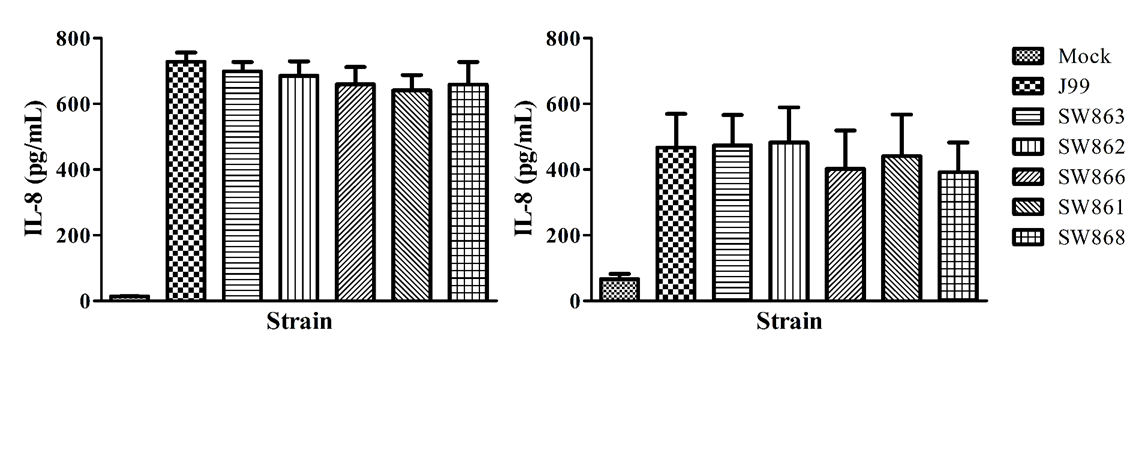
**

**Fig. S9.** ELISA assay for quantifying the IL-8 production in AGS cells (left panel) and GES-1 cells (right panel) infected with the examined strains. Mock = mock infected control. Results are representative of 3 independent experiments (means ± SD). SW863, *jhp0106* mutant; SW862, *jhp0106* revertant; SW866, *flaA* mutant; SW861, *flaB* mutant; SW868, *flaA*/*flaB* mutant.
